# A Descriptive Review on the Potential Use of Diatom Biosilica as a Powerful Functional Biomaterial: A Natural Drug Delivery System

**DOI:** 10.3390/pharmaceutics16091171

**Published:** 2024-09-05

**Authors:** Sunggu Kang, Yeeun Woo, Yoseph Seo, Daehyeon Yoo, Daeryul Kwon, Hyunjun Park, Sang Deuk Lee, Hah Young Yoo, Taek Lee

**Affiliations:** 1 Department of Chemical Engineering, Kwangwoon University, 20 Kwangwoon-ro, Nowon-gu, Seoul 01897, Republic of Korea; rtr2001@kw.ac.kr (S.K.); yenn0706@kw.ac.kr (Y.W.); akdldytpq12@kw.ac.kr (Y.S.); lovydh@kw.ac.kr (D.Y.); andy9760@kw.ac.kr (H.P.); 2 Protist Research Division, Biological Resources Research Department, Nakdonggang National Institute of Biological Resources (NNIBR), 137, Donam 2-gil, Sangju-si 37242, Gyeongsangbuk-do, Republic of Korea; kdyrevive@nnibr.re.kr (D.K.); diatom83@nnibr.re.kr (S.D.L.); 3 Department of Biotechnology, Sangmyung University, 20, Hongjimun 2-gil, Jongno-gu, Seoul 03016, Republic of Korea

**Keywords:** diatom biosilica, drug delivery system, surface functionalization, anticancer drugs, wound healing, bone regeneration

## Abstract

Although various chemically synthesized materials are essential in medicine, food, and agriculture, they can exert unexpected side effects on the environment and human health by releasing certain toxic chemicals. Therefore, eco-friendly and biocompatible biomaterials based on natural resources are being actively explored. Recently, biosilica derived from diatoms has attracted attention in various biomedical fields, including drug delivery systems (DDS), due to its uniform porous nano-pattern, hierarchical structure, and abundant silanol functional groups. Importantly, the structural characteristics of diatom biosilica improve the solubility of poorly soluble substances and enable sustained release of loaded drugs. Additionally, diatom biosilica predominantly comprises SiO_2_, has high biocompatibility, and can easily hybridize with other DDS platforms, including hydrogels and cationic DDS, owing to its strong negative charge and abundant silanol groups. This review explores the potential applications of various diatom biosilica-based DDS in various biomedical fields, with a particular focus on hybrid DDS utilizing them.

## 1. Introduction

Diatoms possess unique porous silica cell walls and are micro-sized algae found in aquatic ecosystems worldwide [1,2]. To date, approximately 18,000 species of diatoms have been identified, which are widely distributed in various environments, including freshwater, brackish water, and oceans [3,4]. Owing to their high adaptability, large-scale deposits of diatomite, comprising diatom remains, are frequently discovered in various geological strata [5,6]. In addition, diatoms have high value as a natural resource for producing biosilica (diatom biosilica) and can be easily cultivated in an artificial environment; therefore, diatoms can be actively employed in a wide range of industrial applications [7,8].

Diatom biosilica (DB) derived from cultures, ecosystems, or diatomite is considered an excellent natural resource for various biomedical applications [9,10,11]. Owing to its three-dimensional hierarchical structure with nanoscale pores and superior physicochemical properties, DB has been applied in numerous fields, including biomedicine and separation technologies [12]. Moreover, DB primarily comprises inorganic silica and, hence, has high biocompatibility owing to its excellent stability. Therefore, accumulated evidence has suggested the potential of DB as a functional biomaterial in drug delivery systems (DDSs) for various purposes, such as anticancer, antibiotic, and hemostatic agents [13].

Various material-based DDS platforms have been developed to improve drug bioavailability [7,14,15]. With increasing reports highlighting the side effects of artificially synthesized drug carriers, DDSs composed of natural resources (e.g., biosilica, cellulose, and alginate) with low toxicity and fewer side effects have garnered considerable interest in the medical and pharmaceutical fields [16,17]. From this perspective, the porous hierarchical structure and high biocompatibility of DB make it a highly effective, sustained-release DDS platform [18]. Moreover, recent studies have actively explored the development of hybrid DDSs by combining DB with other DDS platforms (e.g., lipid nanoparticles (LNP) and hydrogels), leveraging their physicochemical characteristic-based surface modifications [19,20].

In this study, we systematically discuss the characteristics and advantages of DB as a natural resource in the biomedical field, especially in DDS. The Section 2 provides an in-depth analysis of the various properties of DB that make it an effective biomaterial. The Section 3 summarizes the current status of studies on DDS using DB to treat various disease models. The Section 5 discusses the ongoing development of DB-based hybrid DDSs, which is currently the focus of considerable research interest.

## 2. Characteristics of DB

DB can be produced under eco-friendly conditions using diatoms and is considered an excellent biomaterial in the biomedical field [21]. Regarding the physical properties, DB reportedly possesses high biocompatibility, as well as thermal and mechanical stability, owing to the combination of various characteristics, including low thermal conductivity and a uniform porous nanostructure [22,23]. These advantages highlight the potential application of DB as a multifunctional biomaterial. Consequently, this section details the biosynthetic process and properties of DB, focusing on its applications in the biomedical field.

### 2.1. The Mechanism of DB Synthesis

**Figure 1:**
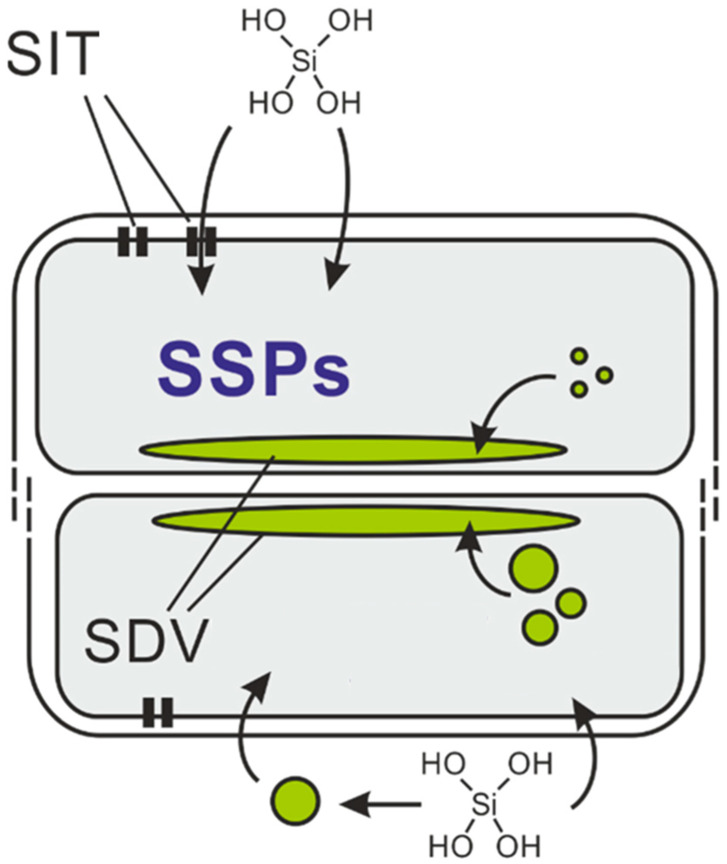
Schematic of silica formation during diatom cell wall synthesis [28].

Diatoms convert absorbed inorganic silicon (mainly orthosilicic acid, Si(OH)_4_) into silica (SiO_2_) through enzymatic reactions, forming silica-based cell walls with the help of proteins and organic molecules, yielding complex-patterned silica structures [24]. This process occurs within specialized compartments called silica deposition vesicles (SDVs), which are crucial for silica biosynthesis and require silicic acid uptake from the environment [25]. Diatoms use silicic acid transporter (SIT) proteins to move Si(OH)_4_ into cells and then into SDVs, where an acidic environment promotes gelation and polymerization [26]. The role of SITs is complex and debated, with other mechanisms like membrane diffusion and endocytosis also considered [27]. Due to low environmental silicic acid levels, diatoms store silicon in a silicon storage pool (SSP) to prevent premature polymerization, which is essential given silica’s low solubility at neutral pH [28]. Proteins such as silaffins and polyamines rapidly precipitate silica [29,30], forming variously sized spheres, with cytoskeletal elements like microtubules and actin regulating SDV movement and shape [31,32] (Figure 1).

### 2.2. The DB Structure

**Figure 2:**
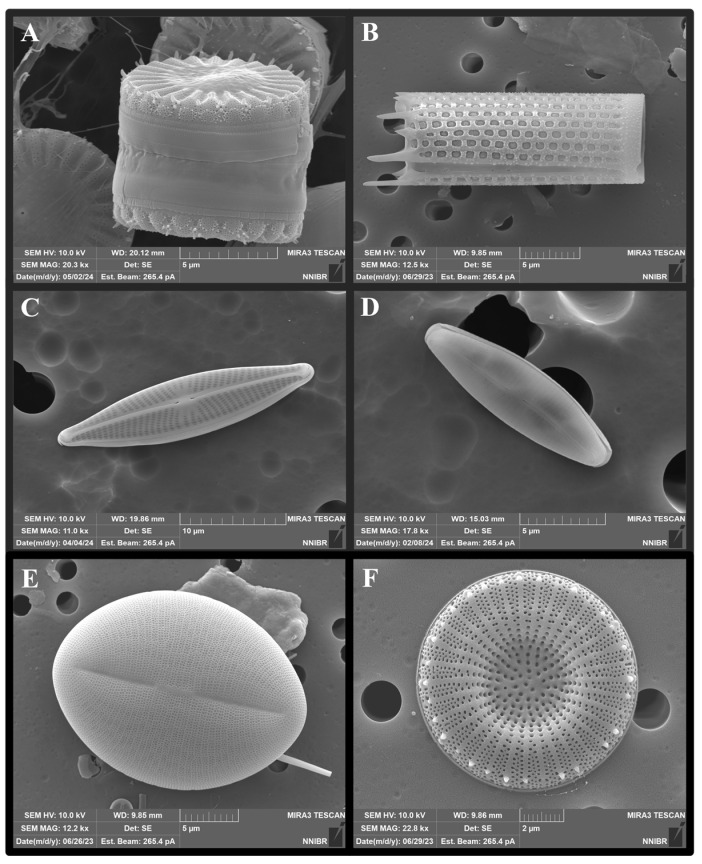
Scanning electron microscope images of biosilica with various structures depending on the species. The dried samples were observed at a magnification of ×2500 to ×15,000 using a field emission scanning electron microscope (FE-SEM) (MIRA 3, TESCAN, Czech Republic) and photographed. (**A**) *Stephanocyclus meneghinianus*, (**B**) *Aulacoseira granulata*, (**C**) *Navicula cryptocephala*, (**D**) *Craticula molestiformis*, (**E**) *Cocconeis placentula*, and (**F**) *Discostella asterocostata*.

The DB surface has a homogenized nanoscale porous structure, while the interior exhibits a complex hierarchical structure [33]. DBs vary in size from a few to several hundred micrometers and possess high durability despite their thin walls, typically ranging from 0.1 to 1 μm in thickness [18,26]. The porous DB structure not only ensures mechanical stability but markedly impacts the sustained release of loaded materials and the adsorption of various ions in aqueous solutions [34,35]. Depending on the species, diatoms can form various shapes, including rod-shaped (e.g., *Thalassionema* sp. and *Synedra* sp.), disc-shaped (e.g., *Actinocyclus* sp. and *Cyclotella* sp.), and cylindrical (e.g., *Aulacoseira* sp. and *Orthoseira* sp.) (Figure 2). The unique structural characteristics of DB, along with its ability to form diverse sizes and shapes, indicate the potential of DB for customized applications tailored to meet specific needs in fields ranging from biotechnology to environmental science [36,37]. These diverse applications underscore the versatility and potential for innovation presented by DB, making it a notable focus of current and future research efforts [9,38,39].

### 2.3. Biocompatibility of DB

In the pharmaceutical field, DB is well-recognized as an effective sustained-release DDS owing to its excellent biocompatibility and porous hierarchical structure [7,18,40]. DB, an amorphous substance composed of SiO_2_, can be decomposed into silica nanoparticles in vivo and is gradually dissolved through a metabolic process and converted into a water-soluble form called Si(OH)_4_. This form is known to avoid the reticuloendothelial system (RES) and does not cause liver toxicity, etc. [41]. The biocompatibility of DB is related to the degradation of biosilica and the biodegradability of silica nanoparticles, which is very important for confirming the potential of DB as a drug delivery vehicle. However, the biodegradability of DB in vivo is yet to be comprehensively established, and this section discusses studies addressing this topic. Shaheer and his team have confirmed the potential of diatoms as drug delivery vehicles due to their structural advantages and high biodegradability [42]. They reduced the size of diatoms by sonication (200–400 nm), and the silica nanoparticles formed from the diatoms were degraded by 60% in PBS at pH 7.4 at 37 °C for 30 days. This degradation suggests that it could lead to sustained release of therapeutic agents in the body, and the increased available surface area of the degraded Si particles could prevent continuous accumulation of the particles. Zhai et al. utilized transmission electron microscopy, confocal microscopy, and inductively coupled plasma–optical emission spectroscopy to examine the distribution and degradation of hollow mesoporous silica nanoparticles (HMSNs) in human umbilical vein endothelial cells (HUVECs). The authors found that HMSNs underwent degradation within the cytoplasm and lysosomes of HUVECs, and degradation products were excreted into the culture medium [43]. These findings indicate that the body can efficiently handle and eliminate silicic acid [44]. Moreover, natural silica (i.e., DB) was found to be more biodegradable and biocompatible than synthetic silica [45,46].

**Figure 3:**
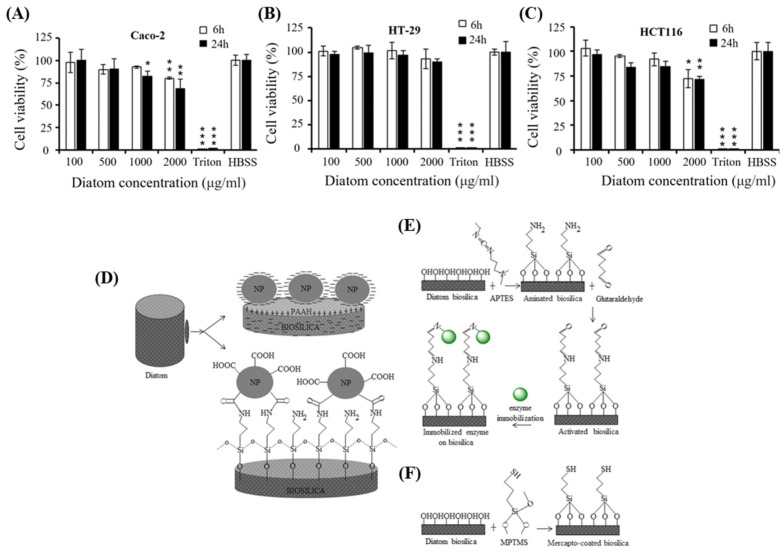
Viability of (**A**) Caco-2, (**B**) HT-29, and (**C**) HCT-116 cells after 6 h (white bars) and 24 h (black bars) incubation with DB microparticles at 37 °C [47]. An unpaired Student’s *t*-test was performed to assess statistical significance. The significance levels were set as follows: * *p* < 0.05, ** *p* < 0.01, and *** *p* < 0.001. (**D**) Schematic of DB and nanoparticle conjugates. (**A**) Layer-by-layer assembly of biosilica and nanoparticles. (**B**) Covalent coupling of biosilica and nanoparticles. (**E**,**F**) Combination of nanoparticles and biosilica via chemical functional groups: (**E**) 3-aminopropyltriethoxysilane (ATPES), (**F**) 3-mercaptopropyl-trimethoxysilane (MPTMS) [12].

Zhang et al. performed in vitro cytotoxicity assays using colon cancer cells (Caco-2/HT-29/HCT116) to verify the cytotoxicity of DB-based microcapsules and their suitability as oral drug carriers (Figure 3A–C) [47]. The formulated microcapsules exhibited low toxicity toward Caco-2/HT-29 cells (up to 1000 μg/mL, for 24 h), thereby confirming the safety of DB as an oral DDS platform. In addition, biosilica has various possibilities for surface modification using nanoparticles or specific functional groups (Figure 3D–F) [12]. Cicco et al. developed a method for chemically modifying the DB surface to generate amino- and mercapto-coated DB-based microcapsules; this was achieved by silanization using 3-aminopropyl-triethoxysilane (APTES) and mercaptopropyl-trimethoxysilane (MPTMS) [40]. Subsequently, cell responses were examined in the human osteosarcoma Saos-2 cell line and normal human dermal fibroblasts. Notably, exposure to DB did not induce any harmful reactions in these cells. This finding highlights the potential of diatom frustules as natural biomaterials that promote cell growth owing to their excellent biocompatibility.

Based on these advantages, DB has considerable potential for designing and developing optimal drug delivery systems (DDSs), offering innovative approaches for treating various disease models. Furthermore, the abundant silanol (Si-OH) groups on DB surfaces can facilitate chemical bonding with a range of substances, including drugs; proteins, such as antibodies and enzymes; and biological components, such as DNA [12,47]. This diverse surface modification capability enables stable drug loading and controlled release from DB, maximizing the drug delivery efficiency by incorporating various functionalities, particularly targeting capabilities [48]. Consequently, DB is now recognized as a natural DDS platform for alternating the synthesis of drug carriers and improving the therapeutic delivery mechanisms of various drugs.

## 3. DB-Based Drug Delivery System

In medical and pharmaceutical fields, numerous studies have explored the development of drugs with various properties for disease treatment [49,50,51]. Extensive efforts have been made to improve drug bioavailability and reduce patient discomfort by incorporating drugs into sustained-release DDSs capable of eliciting improved efficacy [52,53]. The advent of DDSs that can deliver substances encapsulated in micro-sized carriers into the body has drawn substantial global interest owing to their potential to markedly improve drug bioavailability [54,55]. In addition, the use of highly biocompatible drug carriers can reduce patient discomfort and increase patient compliance [56,57].

Natural resources synthesized by organisms are known to exhibit excellent biocompatibility [58,59]. Among these, highly mineralized DB is recognized as a powerful natural DDS owing to its excellent sustained release capabilities and biocompatibility [18,60]. The unique properties of DB, including its nanoporous structure and ability to undergo chemical modification, make it an ideal candidate for drug delivery applications. Furthermore, DB can be engineered to encapsulate diverse therapeutic agents, allowing targeted and controlled drug release that enhances treatment efficacy and minimizes side effects.

However, this field is still in its infancy, and there are still several challenges to be addressed. First, pure DBs lack an intrinsic targeting ability, which may reduce drug efficacy or cause unintended side effects. Some studies have used magnetism to impart a targeting ability to DB-based systems [61], but more commonly, targeting is achieved by functionalizing the DB surface with molecules such as antibodies or ligands [62,63]. However, this surface modification process complicates the synthesis and significantly increases the cost. Second, a major challenge for DB-based drug delivery systems lies in the precise control of drug release rates. Although DBs offer the advantage of sustained release over conventional formulations, they lack the ability to finely tune the release rate in various in vivo environments. To address this, research has focused on functionalizing and controlling the DB surface using polymeric materials. For example, Kumeria et al. developed a pH-sensitive drug release system utilizing DB and graphene oxide (GO) [64]. Surface functionalization with GO demonstrated the potential for pH-responsive release control and maintained a higher sustained release compared to the unmodified control.

In addition, a common problem reported in DB-based drug delivery systems is the rapid release of the drug in the initial phase, known as the “burst release” phenomenon. Various strategies, including DB surface functionalization, are being investigated to alleviate this problem [65]. In conclusion, DB-based drug delivery systems are a promising technology, but continued research is essential to overcome the challenges associated with improving their targeting ability and achieving controlled release rates.

**Figure 4:**
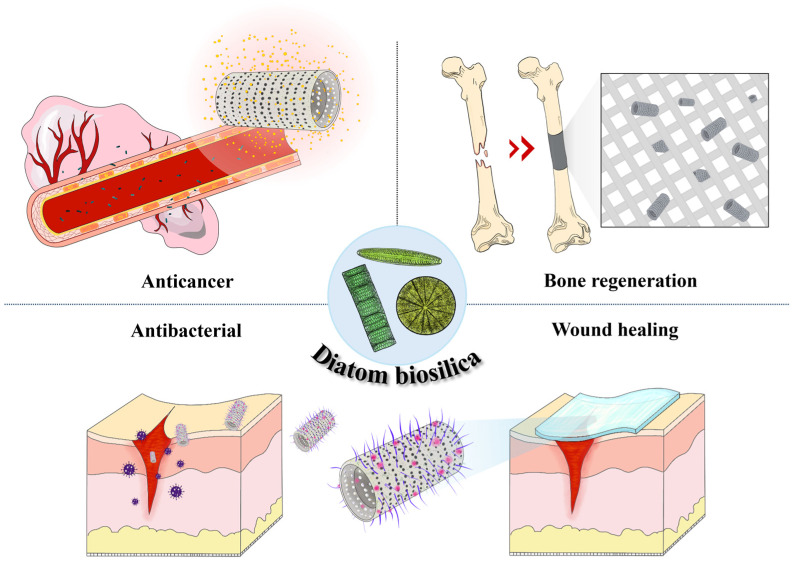
Schematic diagram of the use of a diatom biosilica-based drug delivery system.

This section discusses the current status and advantages of using DB to treat various diseases. The utilization of DB in drug delivery not only leverages its biocompatibility but also its mechanical strength and chemical stability, which are critical for maintaining the integrity of the drug carrier under physiological conditions. Recent studies have revealed promising results using DB-based carriers for cancer therapy [61,66,67,68], anti-inflammatory and antibiotic therapy [69,70,71,72,73,74], bone regeneration [75], and wound healing [76] showcasing their versatility and potential to revolutionize modern medicine (Figure 4) (Table 1).

### 3.1. Anticancer Drug Therapy

In cancer treatment, chemotherapy exerts strong inhibitory effects on cancer cells but also causes severe side effects, such as collateral damage to healthy tissues, owing to a lack of a targeting ability [90,91]. To minimize these side effects, the use of DDSs has been proposed to enhance the bioavailability of drugs by coupling them to drug carriers [92]. In particular, DDSs play a crucial role in current cancer therapy research, as they can effectively address the issue of non-targeting, which remains the main drawback of traditional chemotherapy treatments. This often leads to considerable collateral damage to healthy tissues, along with other severe adverse effects [93,94,95]. Accordingly, the coupling of anticancer drugs with DDSs has been explored as a potential strategy to improve the precision and efficacy of drug delivery, thereby reducing adverse effects and enhancing therapeutic outcomes.

DB has garnered substantial attention owing to its unique structural characteristics, such as its multilayered pore structure. Notably, this distinctive feature inhibits the initial burst release of anticancer drugs, a common issue that can lead to suboptimal therapeutic effects and increased toxicity. Furthermore, chemical treatments can enhance the bio-adhesive properties of DB, improving their bioavailability [96,97].

In addition, several newly developed anticancer drugs are highly hydrophobic, posing challenges to their effective delivery and uniform distribution within the body. In this context, the porous structure of DB has been deemed particularly advantageous, as it facilitates the efficient loading and controlled release of poorly soluble drugs. This capability ensures a more uniform distribution of anticancer agents without the need for harmful solvents, thereby enhancing overall therapeutic efficacy and patient safety [98,99].

For example, Maher et al. employed DB processed via magnesium reduction as a carrier for doxorubicin (DOX) delivery [42]. The authors examined the DB degradation profile in simulated biological environments using inductively coupled plasma mass spectrometry (ICP-MS), revealing that more than 60% of the material degraded within 30 days. The DOX loading efficiency exceeded 94%, with an initial burst release of ~35% within the first 6 h, followed by sustained release over the subsequent 30 days. Based on cell toxicity assay results, treatment with micro-sized carriers up to a concentration of 1000 μg/mL did not induce notable cytotoxicity in RAW 264.7 macrophages and MDM-MB 231-TXSA cells. Furthermore, DB-loaded DOX had a higher drug delivery efficiency than free DOX.

Li’s research team suggested the possibility of targeted drug delivery under the influence of a magnetic field by adsorbing magnetic nanoparticles on the surface of diatoms through electrostatic attraction [61]. The proposed DB-based drug delivery system confirmed that the drug (DOX) loading efficiency was the highest at 29.1% at a diatom concentration of 0.75 mg/mL. The release profile of DOX loaded into DB was investigated in pH 7.4 and 5.0 environments, respectively. In both cases, an initial burst release trend was observed up to 8 h, but stabilization was observed after 10 h, and the cumulative amount of drug released under weakly acidic conditions was higher. This shows that the weakly acidic living environment around cancer cells can induce a rapid drug release rate. The suitability of DB as a drug delivery vehicle was performed through cell experiments, and DB did not significantly affect the viability of normal cells (3T3) and cancer cells (MCF-7) at concentrations of 0.01 mg/mL to 2 mg/mL. In both experimental groups, where DOX was loaded onto DB and DB coated with magnetic nanoparticles, cell viability was 11.16% and 8.33%, respectively. DB coated with magnetic nanoparticles showed successful anticancer effects after reaching target cells beyond the microfluidic channels due to the influence of the magnetic field. Thus, to use diatoms as anticancer drug delivery carriers, proper evaluation of drug target delivery, drug control release, and biotoxicity related to the biodegradability of DB should be performed, and recent studies have focused on the possibility of easy surface functionalization of diatoms.

### 3.2. Anticancer DDS through DB Surface Functionalization

The DB surface, known for its silanol structure, is highly amenable to a range of physicochemical modifications that facilitate extensive surface functionalization [12,100]. This adaptability allows for diverse interactions between drug molecules and DB surface functionalities, enabling precise modifications tailored to specific drug properties. These modifications are crucial for ensuring biocompatibility, optimizing drug loading, and achieving desired drug kinetics [101].

Rea et al. demonstrated the potential of DB for intracellular small interfering RNA (siRNA) delivery [80]. The authors amino-functionalized purified DB using APTES solution, loaded it with siRNA, and subsequently formed complexes by conjugation with peptides to ensure RNAse stability. By grafting siRNA/peptide complexes onto functionalized DB and confirming gene silencing in H1355 cells by Western blotting, the authors highlighted the viability of DB for in vivo siRNA delivery. The study also investigated siRNA release from the loaded DB by measuring the fluorescence intensity over time. The siRNA was released in two phases: an initial burst release over the first 12 h, followed by a slower, sustained release over 48 to 72 h. These findings underscore the potential of DB in enhancing the stability and delivery efficiency of genetic materials, a notable advancement in gene therapy.

**Figure 5:**
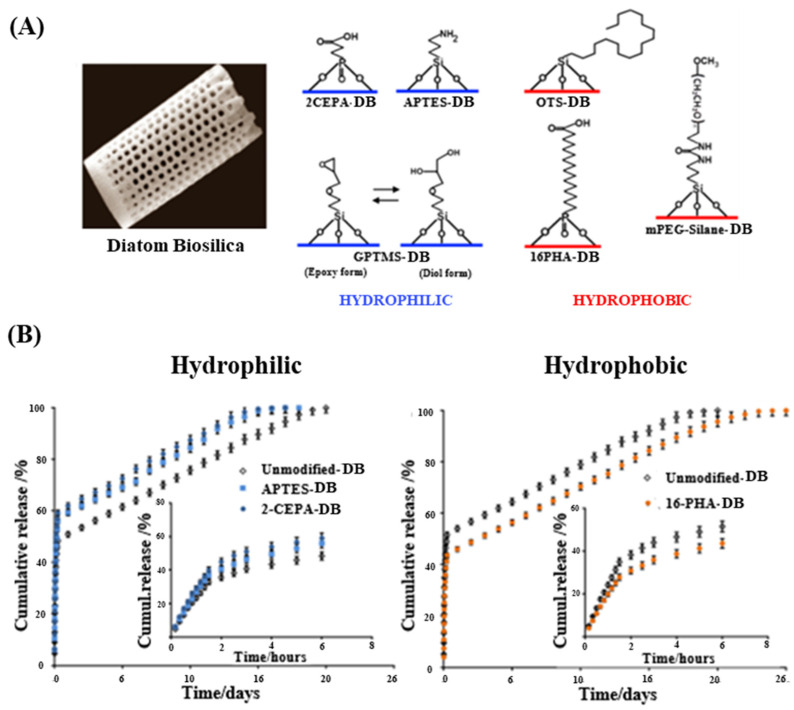
(**A**) Schematic of DB microparticles with surface functionalization using organosilanes (APTES, GPTMS, 7-octadecyltrichlorosilane (OTS), mPEG-Silane) and phosphonic acids (2-carboxyethyl-phosphonic acid (2-CEPA), 16-phosphono-hexadecanoic acid (16-PHA)) to create hydrophilic or hydrophobic surfaces. (**B**) In vitro drug release of gentamicin (GEN) from DB microparticles with hydrophilic and hydrophobic modifications [65].

Bariana et al. adopted a different approach by modifying the surface of DB using self-assembled organic monolayers (SAMs) with four types of silanes: APTES, 3-glycidyloxypropyl trimethoxysilane (GPTMS), trichloro(octadecyl)silane, and methoxy-poly-(ethylene-glycol)-silane (mPEG-silane) [65]. A comprehensive analysis of the drug loading and release kinetics of both hydrophobic (indomethacin, IND) and hydrophilic (gentamicin sulfate, GEN) drugs revealed that surface modification with hydrophilic molecules (APTES, GPTMS, and 2-carboxyethyl-phosphonic acid) can substantially enhance the loading capacity of hydrophobic drugs (Figure 5A). Specifically, 2-carboxyethyl-phosphonic acid-modified DB showed the highest loading capacity for hydrophobic drugs (IND) at 24%, while hydrophobic monolayers led to lower loading capacities (~14%). Conversely, hydrophobic modifications (phosphonic acid and mPEG-silane) not only enhance the capacity for loading hydrophilic drugs on DB but also induce sustained release. The release profiles exhibited an initial burst release followed by a prolonged release over 13~26 days, with hydrophilic surface modifications resulting in slower release rates for hydrophobic drugs. Accordingly, these results provide valuable insights into the importance of surface chemistry in DDSs, demonstrating how tailored surface modifications can improve the drug loading efficiency and release profiles (Figure 5B).

Uthappa et al. developed pH-sensitive drug delivery systems for diclofenac sodium by modifying the surface of diatomite biosilica (DB) using xerogels (XER) [68]. Upon analyzing the drug release kinetics, the authors found that surface modification increased the loading rate from 38.8% in the control group to 46.2%. By altering the internal pore arrangement using xerogels, the authors were able to control drug release, while unmodified DB facilitated controlled release over 16 days, and the surface-modified DB-XER sustained release for 36 days. Additionally, 50.15% of the drug loaded into DB was released within the initial 2 h at pH 1.2, whereas it decreased to 32.45% for the DB-XER sample. This study emphasizes the importance of internal pore structures and surface modifications in prolonging drug release, highlighting the potential for designing and developing more effective and long-lasting DDSs.

**Figure 6:**
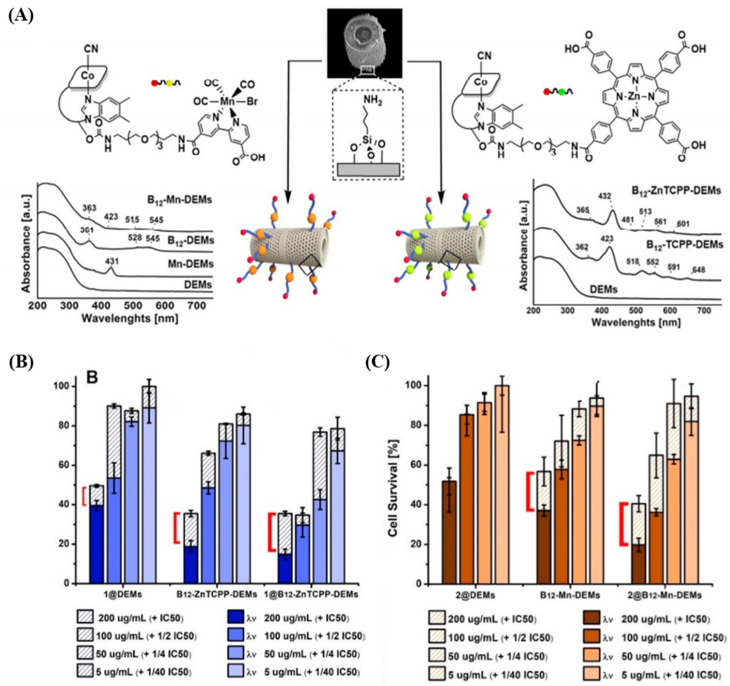
(**A**) Structural formula and SEM images of surface modifications with B12-Mn and B12-Zn/5,10,15,20-Tetrakis (4-Carboxyphenyl) porphyrin (TCPP) complexes, along with solid-state UV–vis spectra confirming the interaction between functionalized DB and tumor mass [68]. (**B**) Histograms depict HCT-116 colorectal cancer cell survival (%) after treatment with varying doses of 1@DEMs, B12-ZnTCPP-DEMs, and 1@B12-ZnTCPP-DEMs, both in the dark (empty bars) and with light activation (filled bars), as well as (**C**) 2@DEMs, B12-Mn-DEMs and 2@B12-Mn-DEMs [63]. (The DEM in this study is the same material as the DB we mentioned).

Delasoie and his research team have successfully demonstrated the controlled and targeted release of anticancer drugs using marine diatoms as a novel drug delivery platform [85]. They proposed a system where diatoms act as micro-shuttles to load highly hydrophobic inorganic anticancer drug complexes, specifically 5-fluorouracil (5-FU) and a tris-tetraethyl [2,2′-bipyridine]-4,4′-diamine–ruthenium(II) (Figure 6A). Within this system, the drug complex remained stable with suppressed release in an aqueous medium for up to 5 days. However, in a lipophilic environment resembling the cellular membrane, more than 95% of the drug was released within 2 h. This characteristic of the system directly relates to the circulation time in the bloodstream, a critical factor for effective targeted cancer therapy, and highlights the potential of the diatom-based micro-shuttle in cancer treatment. Furthermore, the research emphasized the targeting capabilities of cyanocobalamin, a vitamin used by cancer cells, which enabled the diatoms to adhere to colon cancer cells (HT-29) 3 times more effectively than non-targeted diatoms.

Building on this research, Delasoie et al. investigated the combination of vitamin B12 with photoactive molecules on DB (diatomite), followed by surface functionalization to load rhenium(I) tricarbonyl anticancer complexes [63]. Upon light radiation, the functionalized DB targeted colon cancer cells (HCT-116 cells) and doubled the cytotoxicity. Additionally, surface functionalization was maintained under simulated gastric conditions, and the chemical drug groups exhibited a slower release than the control group. This study highlights the potential of developing novel DB-based targeted DDSs utilizing selective photoactivation (Figure 6B,C).

### 3.3. Anti-Inflammatory and Antibiotic Therapy

Inflammation is an immune response triggered by pathogen infection and exposure and can induce the onset of various diseases. In severe cases, inflammation has been known to lead to mortality [102,103,104]. Consequently, in modern medicine, the proper control of inflammation and treatment of diseases rely heavily on anti-inflammatory drugs and antibiotics [105,106]. Notably, anti-inflammatory drugs and antibiotics delivered using DB offer advantages such as sustained release, not only improving patient compliance but ensuring prolonged efficacy and effective treatment with localized administration [47,107]

Zhang et al. explored the potential of DB as an oral anti-inflammatory drug. The authors analyzed the specific surface area and physical properties of DB using N_2_ adsorption and scanning electron microscopy and validated its potential as a DDS through surface chemical modifications before and after drug loading using Fourier transform infrared (FTIR) spectroscopy [47]. Mesalamine and prednisone, common gastrointestinal anti-inflammatory drugs, were loaded onto DB at loading degrees of 11.5 and 9.9 wt.%, respectively. In simulated biological environments, DB facilitated the controlled release of prednisone in gastrointestinal conditions, indicating its potential to enhance drug delivery stability and efficacy.

Vasani et al. designed a thermo-responsive DDS utilizing DB, which was sourced from *Aulacoseria* sp., and surface functionalized it with a silane-based atom transfer radical polymerization (ATRP) initiator [81]. Subsequently, aqueous activators regenerated by electron transfer (ARGET)-ATRP were used to modify the DB surface with thermo-responsive oligo (ethylene glycol) methacrylate polymers. This process resulted in the generation of a thermo-responsive DDS, which was subsequently loaded with the broad-spectrum antibiotic levofloxacin and monitored for gradual drug release. Experiments with Staphylococcus aureus and Pseudomonas aeruginosa confirmed the ability of the drug-loaded DDS to control drug release through temperature modulation. The copolymer had a lower critical solution temperature (LCST) of 36 °C. When the temperature exceeded the LCST, the polymer collapsed, thereby promoting drug release. Conversely, below the LCST, polymer expansion regulated the pore size of the multilayered structure, enabling controlled drug release.

Aw et al. investigated the potential of using diatomaceous silica for oral and implant drug delivery [69]. They selected indomethacin, an anti-inflammatory drug, as a model compound and successfully loaded it onto DB to study its release properties. The results showed that indomethacin was effectively loaded onto the DB with a high drug loading capacity of approximately 22 wt.%, and sustained drug release was observed over two weeks. Pure indomethacin (25 mg and 75 mg) was completely released within 3 h, whereas the indomethacin adsorbed on the surface of the DB exhibited a rapid release during the first 6 h, followed by a slower and more sustained release due to the pores and hollow internal structure of the diatom. In implant-based drug delivery, 36 mg of indomethacin encapsulated in DB was used, corresponding to the dosage used in clinical settings. In this case, the release rate of the encapsulated indomethacin was nearly twice as slow as that of the free drug. However, the release rate progressively slowed over intervals of 6 h, 24 h, and 7 days, extending the total release time to 12 days. Additionally, such an implant system could be effective for local anti-inflammatory therapy, particularly in cases where inflammation persists over a long period. Diatom encapsulation slowed the release rate by 1.5 to 3 times compared to pure indomethacin across various time intervals. DB offers a promising alternative to synthetic silica materials for designing novel solutions in oral and implant drug delivery applications.

Peng et al. designed a nasal drug delivery system for treating allergic rhinitis based on DB. The model drug, budesonide (Bud), a typical rhinitis treatment drug, was loaded, and functionalization was performed through surface modification using polydopamine (PDA) and carboxymethyl chitosan (CMCS) [108]. The CMCS layer provided high adhesion to the nasal mucosa and had an antibacterial effect, which significantly improved the stability and sustained release of the drug. The drug encapsulation of 23.9 ± 2.01% was confirmed, and the difference in the degree of drug release was confirmed depending on the pH. The drug release pattern was confirmed at pH 7.4 and 5.0, and a slower release rate was observed at pH 7.4, suggesting that long-term and sustained drug delivery was possible. The biocompatibility analysis using human skin fibroblast (HSF) cells confirmed cell viability of more than 80% up to a concentration of approximately 1000 μg/mL, and in particular, less than 5% erythrocyte damage due to the PDA and CMCS coatings showed blood compatibility.

### 3.4. Bone Regeneration

The incidence of various bone fractures and diseases, from minor fractures, such as dental caries, to major skeletal segment fractures, has rapidly increased with the growing aging population, necessitating the development of effective bone regeneration therapies [109]. Bone regeneration is a complex process that requires interactions between various cells, growth factors, and vascular networks. Effective bone-regeneration strategies frequently involve the construction of scaffolds using biomaterials and tissue engineering approaches [109,110,111]. Among these strategies, the controlled and targeted release of drugs via DDSs is emerging as one of the most promising methods for enhancing bone regeneration [112,113]. Owing to its application within the body, a DDS for effective bone regeneration warrants high biocompatibility and the ability to achieve a controlled drug release profile [114,115].

DB is a promising DDS for bone regeneration owing to its amorphous nature, high biocompatibility, and ability to facilitate the controlled release of hydrophobic drugs [116,117]. Additionally, natural silica is considered a source of silicon that can stimulate the proliferation and differentiation of osteoblasts, the cells responsible for bone formation [118,119]. Dalgic et al. demonstrated successful osteogenic activity by inducing the controlled release of melatonin (ML) from DB [118]. Recent studies have shown that ML is involved in bone cell proliferation, osteoblast differentiation, and protection [120,121].

**Figure 7:**
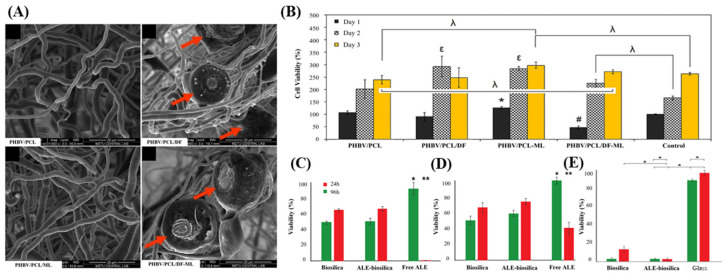
(**A**) SEM images of electrospun fibers at each manufacturing step. The red arrows indicate the DF loaded in the polymer fibers. (**B**) Percent cell viability of Saos-2 cells on scaffolds after 1, 4, and 7 days; denotes the lowest on day 1, the highest on day 1, ε the highest on day 4, and λ statistically different groups (*p* < 0.05) relative to control (TCPS) [118]. # represents the lowest group on day 1, while * indicates the highest group on day 1. (**C**) SaOS-2 and (**D**) BMSC cell viability on biosilica, ALE-biosilica, and free drug solution; significant differences at 24 h (*) and 96 h (**) for *p* < 0.05. (**E**) J774 cell viability on biosilica, ALE-biosilica, and glass control; significant differences for * *p* < 0.05 [83].

However, ML tends to exhibit a rapid release in aqueous media. To address this issue, Dalgic et al. [118] fabricated a functional drug delivery system by electrospinning DB into a polymer solution to attain a polymer-coated delivery system (PHBV/PCL/DF) (Figure 7A). The PHBV/PCL/DF-ML system demonstrated controlled ML release in aqueous media and enhanced the alkaline phosphatase (ALP) activity of SaOS-2 osteosarcoma cells. This improvement was attributed to the combination of DB with PHBV and PCL in biodegradable fibers (PHBV/PCL/DF-ML), which resulted in higher ALP activity than the PHBV/PCL system alone and achieved burst release of the loaded drug. Additionally, treatment with PHBV/PCL/DF retained a high viability of SaOS-2 cells on the scaffold, indicating its potential as a suitable material for bone tissue engineering (Figure 7B).

Mohammadi et al. suggested that scaffolds containing DB could positively impact bone regeneration [122]. Using natural scaffolds composed of gelatin, chitosan, and hyaluronic acid (GCH scaffold) and a DB-incorporated scaffold (GCH-Di scaffold), the authors found that the GCH-Di scaffold achieved the highest bone formation and density in a rat tibial defect model. The biosilica in the scaffolds enhanced the calcification, proliferation, and function of bone cells, thereby promoting the healing process. Consequently, biosilica derived from diatoms was confirmed to enhance scaffold stability and stimulate new bone tissue formation, with the porous structure of DB contributing to its biomineralization potential.

Cicco et al. demonstrated that sodium alendronate (ALE)-loaded DB enhanced osteoblast growth and reduced osteoclast metabolic activity [83]. An analysis of osteoblast viability in SaOS-2 osteosarcoma cells revealed that free ALE environments completely inhibited cell metabolism after 96 h of culture, whereas environments treated with DB and ALE-DB exhibited a gradual increase in SaOS-2 cell density (Figure 7C). Additionally, the effects of ALE-DB have been confirmed in bone marrow stem cells (BMSC), a multipotent progenitor cell line that plays a crucial role in the dynamic equilibrium of bone metabolism (Figure 7D). The inhibitory effect of ALE-DB on osteoclast activity was evaluated in J774 cells (Figure 7E). After 96 h, cells cultured on glass showed no cell death, whereas those in the ALE-DB-treated environment showed 95% cell death (nuclear fragmentation). DB alone resulted in 80% cell death after 96 h. These results indicate that DB can improve the bioavailability of ALE, promote osteoblast differentiation, and inhibit osteoclasts, demonstrating its effectiveness as an osteoconductive biomaterial. Therefore, DB is regarded as an excellent drug delivery material for bone regeneration. However, additional studies are required to elucidate the detailed mechanisms and the in vivo responses.

### 3.5. Wound Healing

Wounds can cause bleeding, excessive inflammation, and secondary infections, which can be life-threatening depending on their severity and, thus, require sustained and complex treatments [123,124]. Additionally, exposed wounds are susceptible to bacterial infections, which, if left untreated for extended periods, can develop into chronic ulcers [125,126]. To prevent these complications, rapid hemostasis, proper isolation of the wound site, and continuous moisture supply are essential [127,128]. Recently, with the growing interest in developing non-compressive hemostatic materials and products, studies have explored the potential of DB, which can quickly absorb large amounts of moisture owing to its highly porous hierarchical structure, as a safe and effective hemostatic agent [89,129].

**Figure 8:**
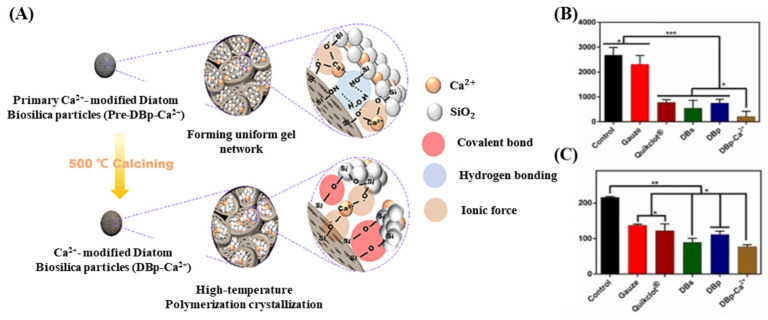
(**A**) Fabrication of Ca^2+^-modified diatom biosilica particles DBp-Ca^2+^ through granulation and sintering process. (**B**) Measurement of blood loss at the rat tail transected site. (**C**) Measurement of clotting time at the rat tail transected site [89]. * indicates a significant difference with *p* < 0.05, ** indicates *p* < 0.01, and *** indicates *p* < 0.001.

Su et al. confirmed that DB can induce rapid blood coagulation owing to its unique structural properties and high capacity to adsorb plasma proteins [89]. The authors modified the DB surface through a granulation process using a calcium ion-containing silica sol. The fabricated DB particle (DBp) Ca^2+^ exhibited high hygroscopicity and blood coagulation properties, and these effective hemostatic properties were attributed to the structural advantages of the hierarchical pores in DB, along with increased interactions between calcium ions and coagulation proteins (Figure 8A). Additionally, DBp Ca^2+^ demonstrated excellent hemostatic performance in terms of efficient moisture absorption, extensive protein adsorption, activation of coagulation proteins, rapid hemostasis, and reinforcement of blood cells, showing faster hemostasis and less blood loss than the commercial hemostatic agent Quikclot^®^ (Teleflex, Wayne, PA, USA) (115 s, 790 mg vs. 86 s, 520 mg) (Figure 8B,C). Upon application for emergency bleeding treatment, biosilica-based hemostatic agents removed necrotic tissue more easily than QuikClot and did not induce secondary bleeding. The authors also proposed that the development of DB-based hemostatic agents with efficient bleeding control presents new possibilities for investigating the coagulation-promoting mechanisms of inorganic hemostatic agents.

According to Wang et al., morphological differences in DB, depending on the diatom species, can influence their hemostatic effects [128]. Upon investigating the hemostatic performance of three diatom species with similar shapes but distinct sizes (*Thalassiosira weissflogii*, *Thalassiosira* sp., and *Cyclotella cryptica*), smaller DB sizes were shown to elicit faster hemostasis and greater liquid absorption owing to their higher surface area. In rodent experiments, all three diatom species had hemostasis times comparable to that of the commercial hemostatic agent (QuikClot^®^), although the smaller species exhibited superior hemostatic effects, with 13 to 50% reduced blood loss in vivo.

In a subsequent study, Wang et al. examined the hemostatic effects of three cylindrical diatom species (*T. weissflogii*, *Thalassiosira* sp., and *C. cryptica*) and four pennate diatom species (*Cocconeiopsis orthoneoides*, *Navicula avium*, *Navicula* sp., and *Pleurosigma indicum*). Pennate DB reduced the in vitro coagulation time by approximately 32.4% when compared with QuikClot^®^. In in vivo rat experiments, these DBs showed a 50% reduction in the hemostasis time, along with less blood loss (61% to 73.4%) than the commercial product [130].

Sun et al. developed a hydrophilic bio-polysaccharide adhesive (BSP) and a DB-based composite wound-sealing hemostatic agent (BSP/DB) that mimicked the strong bioadhesive substances (extracellular polymers, EPS) secreted by the attached diatoms [131]. To mimic diatom EPS, BSP, composed of glucose, mannose, and galactose, was mixed with DB. The adhesive strength of the prepared bioadhesive BSP/DB was attributed to enhanced crosslinking promoted by the porous diatomic structure and increased hydrogen bond formation owing to the abundant SiOH- in DB. In ex vivo bullfrog heart perfusion model-based wound closure tests, BSP/DB exhibited improved hemostatic effects and effectively sealed the heart wounds. Additionally, in in vivo rat liver bleeding model experiments, the application of BSP/DB enabled rapid wound closure with less bleeding than the application of the control and BSP materials. In L929 cell assays, cells cultured with BSP/DB exhibited more than 80% cell viability, and the hemolysis tests revealed a hemolysis rate of less than 5%. Consequently, DB-based hemostatic agents demonstrate excellent hemostatic effects and high biocompatibility.

## 4. Secondary DDS: The Use of Lipid Nanoparticles (LNP) and Hydrogels

**Figure 9:**
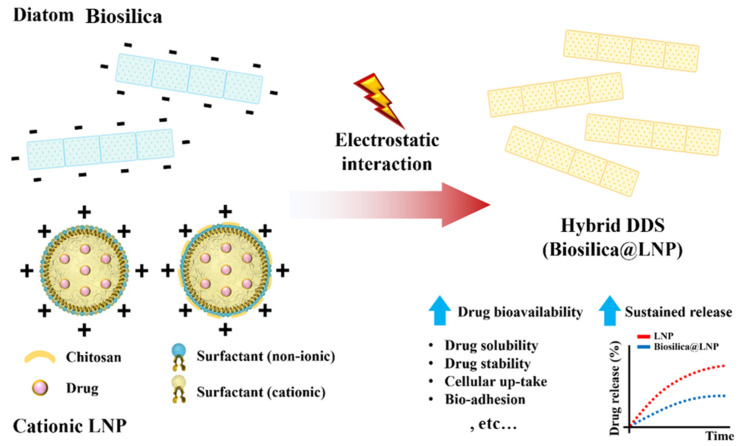
Schematic image of biosilica@lipid nanoparticle hybrid DDS based on electrostatic interaction.

Traditional research on DB-based DDS has predominantly focused on surface modifications for drug loading and carrier functionalization [23,132,133]. These efforts have highlighted the capabilities of DB in enhancing drug stability, delivery efficiency, and controlled release. Recently, there has been a surge in investigations focusing on the development of hybrid DDSs that combine DB with other delivery systems. This hybridization is driven by the abundant functional groups and strong charge characteristics of the DB surface [134,135]. These natural porous silica particles may be capable of addressing the limitations of existing DDSs, such as their low drug-loading capacity and poor control over drug release profiles. The large surface area and unique structural properties of DBs provide a platform for enhancing these aspects. By integrating DB with other DDS technologies, it is possible to create hybrid systems that offer superior performance in drug delivery applications [136,137]. Thus, this section examines the benefits and current progress in developing major hybrid DDS platforms based on DB, emphasizing their innovative potential and transformative impact on modern medicine (Figure 9) (Table 2).

### 4.1. Electrostatic Interaction (e.g., Cationic LNPs and Fe^3+^/Dopamine Complex)

DB, which primarily comprises SiO_2_, has a strong negative charge at neutral pH [34]. This characteristic facilitates the binding of positively charged drugs or substances via electrostatic interactions [143]. For instance, studies have explored the potential of developing carriers that respond to external magnetic fields by conjugating iron oxide nanoparticles with dopamine, which possesses a cationic anchor (amine group), and then attaching them to the DB [77,144]. Recent studies have suggested that neutralizing or replacing the strong negative surface charge of a carrier with a positive charge can enhance its interaction with negatively charged cells (particularly cancer cells), thereby promoting cellular uptake (endocytosis) [145,146]. In addition, the particle size and surface characteristics of drug delivery carriers are crucial parameters that impact the efficiency of drug delivery. The surface charge (or zeta potential) of a carrier is directly related to its interactions with the cell membrane [147]. Therefore, loading cationic drug carriers onto strongly negatively charged DB could potentially overcome issues related to low drug protection, drug-loading capacity, and intracellular drug delivery efficiency.

LNPs, which are widely used in DDS research, can form a strong positive charge on their surfaces with cationic surfactants or chitosan coatings, making them suitable for hybridization with DB [148,149]. LNPs are lipid-based nanoparticles with nanometer-sized dimensions that offer high biocompatibility and biodegradability, thereby minimizing their cytotoxicity [150,151]. Delalat et al. first proposed the use of a DB@LNP hybrid DDS for delivering hydrophobic anticancer drugs [62]. The authors employed cationic LNPs prepared using cationic surfactants to load the hydrophobic drug camptothecin (CPT) and bound it to DB through electrostatic interactions. The DB functioned as a secondary carrier, safely delivering the LNPs to the target organs, whereas biosilica conjugated with antibodies effectively delivered the cationic LNPs to the target cells. Although high nonspecific cytotoxic responses were observed, owing to the use of cationic surfactants alone, using chitosan-coated cationic LNPs could enhance bioadhesiveness, owing to the formation of the chitosan gel layer upon moisture absorption at the target site. This could improve the targeting efficiency of hybrid DDSs [152,153].

### 4.2. Secondary DDS (with Hydrogel)

According to recent studies on DDS, the high-water absorption rate of DB facilitates its hybridization with hydrogels comprising hydrophilic polymer networks [138,154]. Hydrogels, a common type of DDS, are three-dimensional polymer networks synthesized through physicochemical bonds between hydrophilic polymers and include polymers, proteins, small molecules, or colloids, which offer high biocompatibility [155,156].

However, hydrogels tend to become excessively swollen when exposed to excess water, which reduces their mechanical stability. This can lead to uncontrolled drug release or reduced adhesion to the treatment sites, thereby lowering the overall efficiency of drug delivery [157,158]. Hydrogels also exhibit a poor capacity for dissolving hydrophobic drugs, which limits their drug delivery effectiveness [159,160]. DB can enhance the stability of chemical bonds within hydrogels and load hydrophobic drugs through its surface functional groups. Owing to these benefits, research on the development and application of a hydrogel@DB hybrid DDS (HY-DB) is ongoing. HY-DB is used in skin regeneration research because it can maintain moisture, absorb tissue fluids, provide breathability, block infection risks at wound sites, and enhance self-healing [141,161].

Cao et al. proposed a hydrogel–biosilica composite (H/D) that mimicked the extracellular matrix [141]. The authors suggested that DB could strengthen the mechanical properties of the hydrogel; gradually release Si to promote inflammation modulation, angiogenesis, and collagen deposition in chronic wounds; and facilitate the transition from the inflammation phase to the proliferation and remodeling phases. In cell experiments using mouse vascular endothelial cells (C166), the HY-DB-treated group showed a 1.96-fold increase in tube length when compared with the control group, indicating the substantial angiogenic potential of Si released from HY-DB.

**Figure 10:**
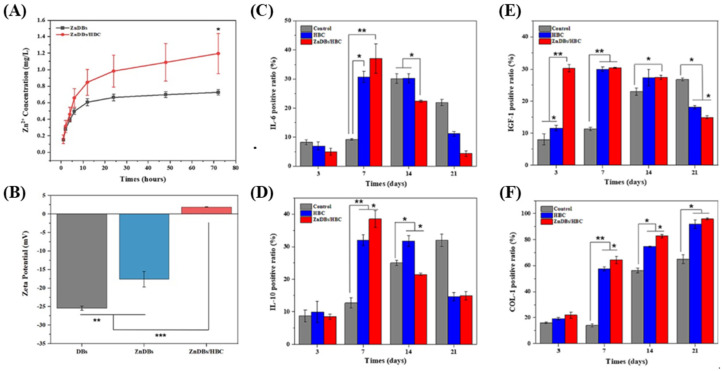
(**A**) In vitro Zn^2+^ release profiles of ZnDBs and ZnDBs/HBC composite hydrogel determined by ICP-AES, depicted as mean ± SD (n = 3); * *p* < 0.05. (**B**) Zeta potential measurements for DBs, ZnDBs, and ZnDBs/HBC composite hydrogel, shown as mean ± SD (n = 3); ** *p* < 0.01, *** *p* < 0.001. (**C**–**F**) Immunohistochemical staining and quantitative analysis for control, HBC hydrogel, and ZnDBs/HBC composite hydrogel groups at days 3, 7, 14, and 21, with data as mean ± SD (n = 3); * *p* < 0.05, ** *p* < 0.01. (**C**,**D**) Analysis of IL-6 and IL-10 cytokines during the inflammatory phase. (**E**) IGF-1 quantification during the proliferative phase. (**F**) COL-1 quantification during the remodeling phase [20].

Ding et al. formulated HY-DB@hydroxylbutyl chitosan (HBC) by attaching zinc to the surface of *Cyclotella cryptica* DB through hydrothermal synthesis and used it to prepare an HBC hydrogel [20]. The authors demonstrated that HY-DB maintained sufficient moisture at the lesion site and slowly released Zn^2+^ to treat inflammation (Figure 10A). Using SEM, energy dispersive X-ray spectrometer (EDX), zeta potential, Fourier transform infrared spectroscopy (FT-IR), and ^1^H nuclear magnetic resonance (NMR) analyses, the researchers successfully characterized the microstructure, chemical interactions, and surface charge changes of ZnDBs with various zinc ratios and the ZnDB/HBC composite hydrogel. In the study, the DB exhibited a zeta potential of −25.4 ± 0.55 mV due to the silanol groups on its surface. The interaction between the amino group of HBC and the silanol groups, along with the shielding of the Zn^2+^ cations, resulted in a zeta potential of +1.86 ± 0.04 mV for the ZnDB/HBC composite hydrogel (Figure 10B). These findings confirm the successful mineralization of zinc and the formation of the composite hydrogel. They detected and quantified the expression of various factors through immunohistochemistry to evaluate the impact of the composite hydrogel at different stages of wound healing. The pro-inflammatory cytokine IL-6 (Figure 10C) and the anti-inflammatory cytokine IL-10 (Figure 10D), which are primarily present in the early stages of wound occurrence, were found to be 3 times and 4 times higher in the HBC and ZnDBs/HBC groups, respectively, compared to the control group on day 7. Additionally, on day 14, while the control group had entered the inflammatory infiltration stage, inflammation in the hydrogel groups had decreased. IGF-1 (Figure 10E), which is involved in cell proliferation and differentiation, showed higher expression levels in the composite hydrogel groups from day 3 to day 14, but these levels decreased compared to the control group from day 14 to day 21. This is interpreted as a result of the rapid wound healing effect of the hydrogel groups. Proper collagen accumulation during the wound remodeling stage promotes tissue repair. From day 7 to day 21, higher expression levels of collagen (COL-1) were observed in the composite hydrogel groups, with no scar formation reported after remodeling (Figure 10F). Consequently, the DB-based composite hydrogel can influence the expression levels of various factors during the wound healing process, confirming its potential as a new wound dressing for the treatment of chronic diabetic wounds.

**Figure 11:**
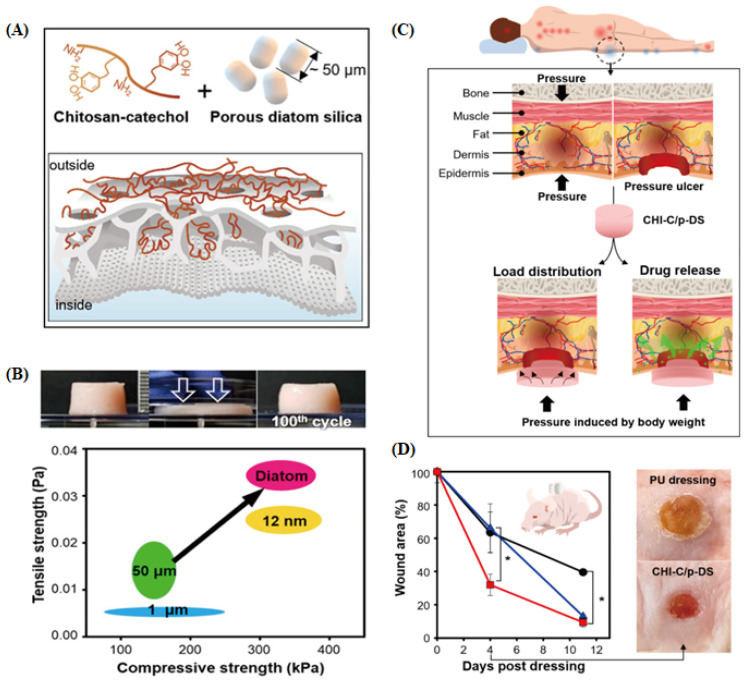
(**A**) Schematic illustration of hydrogel mechanics enhancement through double network cross-linking with natural porous diatom frustule silica. (**B**) Tensile and compressive strength of CHI-C gel with p-DS. (**C**) Application of CHI-C/p-DS elastomeric dressing for pressure ulcer prevention and healing. (**D**) Wound closure in a magnet-induced pressure ulcer model: no dressing (black), PU dressing (blue), CHI-C/DS dressing (red). Photos taken on day 4 [140]; significant differences for * *p* < 0.05.

Lee et al. confirmed the potential of HY-DB as a treatment for ulcerative wounds (Figure 11A) [140]. The elastomer HY-DB-based composite wound dressing showed sustained moisture supply to the wound site, excellent biocompatibility, superior hemostatic effects, antibacterial properties, and controlled drug release. It also exhibited improved physical and mechanical properties, with a 60% deformation rate and up to 400% stretchability, compared with conventional hydrogels (Figure 11B). In rat pressure ulcer models (Figure 11C), the catechol conjugated chitosan and porous diatom silica (CHI-C/p-DS) gel patch, prepared from the hydrogel, reduced the wound area to 31.95 ± 6.46% on day 4, indicating faster healing than the general exposure model (Figure 11D). This suggests that the elastomeric gel patch comprising DB particles and hydrogel can be applied as a wound dressing for patients with pressure ulcers owing to its superior compressibility and energy dissipation properties. Currently, HY-DB is actively used in the field of wound healing, and research on hybrid DDSs combining biosilica and primary drug carriers is ongoing. Because previous studies have mainly focused on wound healing, there is a need for additional research on various administration routes via different physicochemical modifications.

## 5. Conclusions

In the biomedical field, efforts to develop new drugs for treating various diseases are ongoing, with the introduction of DDS aimed at mitigating side effects and improving drug bioavailability. This study proposes a new research direction in biomedical applications by exploring the potential of a hybrid DDS that combines DB with other DDS platforms, moving beyond traditional DDS research that has focused on DB as a single material.

The uniform nanopatterned porous hierarchical structure and superior biocompatibility of DB have led to its recognition as an excellent excipient for achieving a sustained-release DDS platform. Diatoms, which thrive in nearly all aquatic ecosystems, can be readily cultured at various scales with simple media, allowing for the sustainable production and supply of DB in an eco-friendly manner. Additionally, the numerous functional groups (e.g., –OH and silanol) present on the DB surface facilitate hybridization with other DDS platforms (e.g., LNPs and hydrogels) through various physicochemical techniques. The resulting DB-based hybrid DDS demonstrated superior drug delivery capabilities when compared with the individual use of each carrier. In particular, the combination with existing DDS platforms, such as hydrogels and lipid nanoparticles, enhances DB’s biocompatibility and functionality, offering a novel direction for building more efficient drug delivery systems.

However, several challenges need to be overcome for DB to show its inherent value as a natural DDS platform. Diatom biosilica-based drug delivery systems are recognized for their capability to facilitate the sustained release of conventional drugs over time. Despite this advantage, these systems often experience a significant initial burst release of the drug, which can undermine the controlled release profile. Additionally, achieving precise control over drug release in diverse environmental conditions remains a complex challenge that necessitates further in-depth research.

Furthermore, long-term clinical data based on rigorous in vitro and in vivo tests are essential to ensure the stability and cytotoxicity of DB under physiological conditions. Addressing these issues through ongoing research and development efforts will not only demonstrate the superior performance of DB in the biomedical field but also highlight the potential of other natural resource-based DDS platforms.

## Figures and Tables

**Table 1:** Current status of drug delivery using biosilica surface functionalization.

Diatom Species	Drugs	Surface Modification	Effect	References
*Aulacoseira* sp.	Indomethacin	Dopamine-iron oxide	Anti-inflammatory	[77]
*Aulacoseira* sp.	Gentamicin and indomethacin	Hydrophobic/Hydrophilic silane	Anti-inflammatory	[65,78]
*Aulacoseira* sp.	Indomethacin	Graphene oxide	Anti-inflammatory	[64]
Diatomite	Prednisone and mesalamine	-	Anti-inflammatory	[47]
Diatomite	Carbamazepine	SEEPS (Solid self-emulsifying phospholipid suspension)	Psychomotor seizures and trigeminal neuralgia	[79]
Diatomite	siRNA	-	Anti-cancer	[80]
*Aulacoseira* sp.	Levofloxacin	oligo(ethylene glycol)methacrylate	Anti-inflammatory	[81]
*Nitzschia* sp.	Curcumin	CMDM- F and CMDM-I	Antibiotic	[82]
*Thalassiosira weissfloggi* sp.	Ciprofloxacin	APTES-TEMPO (2,6,6,tetramethylpiperidine N-oxy)	Bone growth	[83,84]
Diatomite	Doxorubicin	Magnesium thermal reduction	Anti-cancer	[43]
*Aulacoseira* sp.	Diclofenac sodium (DS)	Xerogel	Antibiotic	[68]
Diatomite, *Coscinodiscus*	Anticancer drugs	cobalamin (vitamin B_12_)	Anti-cancer	[63,85,86]
*Amphora subtropica*	Doxorubicin	Chitosan	Anti-cancer	[87]
*Thalassiosira weissflogii*, *Thalassiosira* sp., *Cyclotella cryptica*	-	-	Hemostasis	[88]
*C. cryptica*	-	Ca^2+^	Hemostasis	[89]

**Table 2:** Current status of drug delivery system research using diatom hybrid composite.

Composite	Type	Target	Effect	References
Diatom/Hydroxybutyl chitosan + Zn^2+^	Hydrogel dressing + Drug delivery	Diabetic chronic wound healing	Sustained release, hemostasis, wound healing, biocompatibility	[21]
Diatom/Hydroxybutyl chitosan + Doxycyclin	Hydrogel dressing + Drug delivery	Wound closure	Sustained release, antimicrobial, hemostasis, biocompatibility, nonadherent	[138]
Diatom/Hydroxybutyl chitosan + Si	Hydrogel dressing + Drug delivery	Chronic wound healing	Sustained release, reduced inflammation, angiogenesis, biocompatibility, collagen deposition	[139]
Diatom/Catechol conjugated chitosan	Hydrogel dressing	Wound dressing for pressure ulcers	Sustained release, reinforced mechanical properties, wound healing	[140]
Diatom/Hydroxybutyl chitosan	Hydrogel dressing	Wound dressing	Reinforced mechanical properties, blood compatibility, thermal stability, biodegradability, biocompatibility	[141]
Diatom/Chitosan/Dopamine	Beads	Hemostasis	Biocompatibility, non-cytotoxicity, rapid hemostasis, enhanced absorption	[142]
Diatom/Polymer coating + Melatonin Diatom/Gelatin/Chitosan/Hyaluronic acid/β-sitosterol Diatom/antibody/Liposome	Scaffold Scaffold Targeted drug delivery	Bone regeneration Bone regeneration Anticancer	Controlled release, increased ALP activity, enhanced osteoblast-like cell viability, enhanced porosity, biodegradability, biomineralization, anti-inflammatory, angiogenic effect natural drug carriers, biocompatible, biodegradable, surface modification, high drug loading (%)	[63,118,122]

## Data Availability

Data sharing is not applicable to this article.

## References

[1] Kociolek J.P. (2018). A worldwide listing and biogeography of freshwater diatom genera: A phylogenetic perspective. Diatom Res..

[2] Wang J., Soininen J., Heino J. (2021). Ecological Indicators for Aquatic Biodiversity, Ecosystem Functions, Human Activities and Climate Change. Ecol. Indic..

[3] Jeong Y., Lee J. (2024). Comparative analysis of organelle genomes provides conflicting evidence between morphological similarity and phylogenetic relationship in diatoms. Front. Mar. Sci..

[4] Li F., Beardall J., Gao K. (2018). Diatom performance in a future ocean: Interactions between nitrogen limitation, temperature, and CO2-induced seawater acidification. ICES J. Mar. Sci..

[5] Wiemer G., Dziadek R., Kopf A. (2017). The Enigmatic Consolidation of Diatomaceous Sediment. Mar. Geol..

[6] Zuluaga-Astudillo D., Ruge J.C., Camacho-Tauta J., Reyes-Ortiz O., Caicedo-Hormaza B. (2023). Diatomaceous Soils and Advances in Geotechnical Engineering—Part, I. Appl. Sci..

[7] Terracciano M., De Stefano L., Rea I. (2018). Diatoms Green Nanotechnology for Biosilica-Based Drug Delivery Systems. Pharmaceutics.

[8] Ghobara M., El-Sheekh M., Hamed A.F., Abdelhamid M.A.A., Pack S.P. (2023). Diatom Nanostructured Biosilica. Value-Added Products from Algae: Phycochemical Production and Applications.

[9] Rabiee N., Khatami M., Jamalipour Soufi G., Fatahi Y., Iravani S., Varma R.S. (2021). Diatoms with Invaluable Applications in Nanotechnology, Biotechnology, and Biomedicine: Recent Advances. ACS Biomater. Sci. Eng..

[10] De Tommasi E., De Luca A.C. (2022). Diatom Biosilica in Plasmonics: Applications in Sensing, Diagnostics and Therapeutics. Biomed. Opt. Express.

[11] Singh P., Srivastava S., Singh S.K. (2019). Nanosilica: Recent Progress in Synthesis, Functionalization, Biocompatibility, and Biomedical Applications. ACS Biomater. Sci. Eng..

[12] Panwar V., Dutta T. (2019). Diatom Biogenic Silica as a Felicitous Platform for Biochemical Engineering: Expanding Frontiers. ACS Appl. Bio Mater..

[13] Dhanker R., Singh P., Sharma D., Tyagi P., Kumar M., Singh R., Prakash S. (2023). Diatom Silica a Potential Tool as Biosensors and for Biomedical Field. Insights into the World of Diatoms: From Essentials to Applications.

[14] Lim H., Seo Y., Kwon D., Kang S., Yu J., Park H., Lee S.D., Lee T. (2023). Recent Progress in Diatom Biosilica: A Natural Nanoporous Silica Material as Sustained Release Carrier. Pharmaceutics.

[15] Gnanamoorthy P., Anandhan S., Prabu V.A. (2014). Natural Nanoporous Silica Frustules from Marine Diatom as a Biocarrier for Drug Delivery. J. Porous Mater..

[16] Jahangirian H., Lemraski E.G., Webster T.J., Rafiee-Moghaddam R., Abdollahi Y. (2017). A Review of Drug Delivery Systems Based on Nanotechnology and Green Chemistry: Green Nanomedicine. Int. J. Nanomed..

[17] Kanwar R., Rathee J., Salunke D.B., Mehta S.K. (2019). Green Nanotechnology-Driven Drug Delivery Assemblies. ACS Omega.

[18] Uthappa U.T., Brahmkhatri V., Sriram G., Jung H.-Y., Yu J., Kurkuri N., Aminabhavi T.M., Altalhi T., Neelgund G.M., Kurkuri M.D. (2018). Nature Engineered Diatom Biosilica as Drug Delivery Systems. J. Control. Release.

[19] Pu Y., Wei M., Witkowski A., Krzywda M., Wang Y., Li W. (2020). A Hybrid Biomaterial of Biosilica and C-Phycocyanin for Enhanced Photodynamic Effect towards Tumor Cells. Biochem. Biophys. Res. Commun..

[20] Ding Y., Mu Y., Hu Y., Liu J., Su C., Sun X., Chen X., Jia N., Feng C. (2024). Zinc-Mineralized Diatom Biosilica/Hydroxybutyl Chitosan Composite Hydrogel for Diabetic Chronic Wound Healing. J. Colloid Interface Sci..

[21] Kim Y., Shin H.A., Choi J.W., Kim M.Y., Go G.M. (2022). Compositional characteristics of the Microalga Melosira nummuloides Mass-cultured using Jeju lava seawater. Korean J. Fish. Aquat. Sci..

[22] Saoud H.A.A.L., Sprynskyy M., Pashaei R., Kawalec M., Pomastowski P., Buszewski B. (2022). Diatom Biosilica: Source, Physical-Chemical Characterization, Modification, and Application. J. Sep. Sci..

[23] Delasoie J., Zobi F. (2019). Natural Diatom Biosilica as Microshuttles in Drug Delivery Systems. Pharmaceutics.

[24] Mayzel B., Aram L., Varsano N., Wolf S.G., Gal A. (2021). Structural evidence for extracellular silica formation by diatoms. Nat. Commun..

[25] Kolbe F., Brunner E. (2022). Silicic Acid Uptake and Storage by Diatoms. The Molecular Life of Diatoms.

[26] Hildebrand M. (2008). Diatoms, Biomineralization Processes, and Genomics. Chem. Rev..

[27] Oa T. (2008). Silicon Uptake in Diatoms Revisited A Model for Saturable and Nonsaturable Uptake Kinetics and the Role of silicon transporters. Plant Physiol..

[28] Reichelt T., Bode T., Jordan P., Brunner E. (2023). Towards the Chemical Analysis of Diatoms Silicon Storage pools: A differential centrifugation-based separation approach. Minerals.

[29] Kröger N., Deutzmann R., Bergsdorf C., Sumper M. (2000). Species-Specific Polyamines from Diatoms Control Silica Morphology. Proc. Natl. Acad. Sci. USA.

[30] Patwardhan S.V., Clarson S.J. (2002). Silicification and Biosilicification: Part 4. Effect of Template Size on the Formation of Silica. J. Inorg. Organomet. Polym..

[31] Van de Meene A.M.L., Pickett-Heaps J.D. (2002). Valve Morphogenesis in the Centric Diatom Proboscia Alata Sundstrom. J. Phycol..

[32] Van De Meene A.M.L., Pickett-Heaps J.D. (2004). Valve Morphogenesis in the Centric Diatom Rhizosolenia Setigera (Bacillariophyceae, Centrales) and Its Taxonomic Implications. Eur. J. Phycol..

[33] Cvjetinovic J., Luchkin S.Y., Davidovich N.A., Bedoshvili Y.D., Salimon A.I., Korsunsky A.M., Gorin D.A. (2023). Characterization of diatom silica exoskeletons using atomic force microscopy: Topography and mechanical properties. Mater. Today Proc..

[34] Qi Y., Wang J., Wang X., Cheng J.J., Wen Z. (2017). Selective Adsorption of Pb (II) from Aqueous Solution Using Porous Biosilica Extracted from Marine Diatom Biomass: Properties and Mechanism. Appl. Surf. Sci..

[35] Vona D., Cicco S.R., la Forgia F.M., Vacca M., Porrell A., Caggiano G., Angelis M.D., Gesualdo L., Farinola G.M. (2024). All Bio-Based µ-Beads from Microalgae for Probiotics Delivery. Adv. Sustain. Syst..

[36] Konak B.M.K., Bakar M.E., Ahan R.E., Özyürek E.U., Dökmeci S., Şeker U.Ö.Ş. (2022). A living material platform for the biomineralization of biosilica. Mater. Today Bio..

[37] Min K.H., Kim D.H., Shin J.W., Ki M.R., Pack S.P. (2024). Microalgae-derived peptide with dual-functionalities of silica deposition and antimicrobial activity for biosilica-based biomaterial design. Process Biochem..

[38] Sharma N., Simon D.P., Diaz-Garza A.M., Fantino E., Messaabi A., Meddeb-Mouelhi F., Germain H., Desgagné-Penix I. (2021). Diatoms Biotechnology: Various Industrial Applications for a Greener Tomorrow. Front. Mar. Sci..

[39] Mishra M., Arukha A.P., Bashir T., Yadav D., Prasad G.B.K.S. (2017). All New Faces of Diatoms: Potential Source of Nanomaterials and Beyond. Front. Microbiol..

[40] Cicco S.R., Vona D., Gristina R., Sardella E., Ragni R., Presti M.L., Farinola G.M. (2016). Biosilica from Living Diatoms: Investigations on Biocompatibility of Bare and Chemically Modified Thalassiosira Weissflogii Silica Shells. Bioengineering.

[41] Ehlerding E.B., Chen F., Cai W. (2016). Biodegradable and Renal Clearable Inorganic Nanoparticles. Adv. Sci..

[42] Maher S., Kumeria T., Wang Y., Kaur G., Fathalla D., Fetih G., Santos A., Habib F., Evdokiou A., Losic D. (2016). From The Mine to Cancer Therapy: Natural and Biodegradable Theranostic Silicon Nanocarriers from Diatoms for Sustained Delivery of Chemotherapeutics. Adv. Healthc. Mater..

[43] Zhai W., He C., Wu L., Zhou Y., Chen H., Chang J., Zhang H. (2012). Degradation of Hollow Mesoporous Silica Nanoparticles in Human Umbilical Vein Endothelial Cells. J. Biomed. Mater. Res. Part B Appl. Biomater..

[44] Anglin E.J., Cheng L., Freeman W.R., Sailor M.J. (2008). Porous Silicon in Drug Delivery Devices and Materials. Adv. Drug Deliv. Rev..

[45] Maher S., Kumeria T., Aw M.S., Losic D. (2018). Diatom Silica for Biomedical Applications: Recent Progress and Advances. Adv. Healthc. Mater..

[46] Chianese C.T.G., Terracciano M., de Stefano L., Rea I. (2020). Nanostructured Biosilica of Diatoms: From Water World to Biomedical Applications. Appl. Sci..

[47] Zhang H., Shahbazi M.-A., Mäkilä E.M., da Silva T.H., Reis R.L., Salonen J.J., Hirvonen J.T., Santos H.A. (2013). Diatom Silica Microparticles for Sustained Release and Permeation Enhancement Following Oral Delivery of Prednisone and Mesalamine. Biomaterials.

[48] Fenton O.S., Olafson K.N., Pillai P.S., Mitchell M.J., Langer R. (2018). Advances in Biomaterials for Drug Delivery. Adv. Mater..

[49] Irrechukwu O., Yeager R., David R., Ekert J., Saravanakumar A., Choi C.K. (2023). Applications of Microphysiological Systems to Disease Models in the Biopharmaceutical Industry: Opportunities and Challenges. ALTEX-Altern. Anim. Exp..

[50] Sahlgren C., Meinander A., Zhang H., Cheng F., Preis M., Xu C., Salminen T.A., Toivola D., Abankwa D., Rosling A. (2017). Tailored Approaches in Drug Development and Diagnostics: From Molecular Design to Biological Model Systems. Adv. Healthc. Mater..

[51] Zitter R., Chugh R.M., Saha S. (2022). Patient Derived Ex-Vivo Cancer Models in Drug Development, Personalized Medicine, and Radiotherapy. Cancers.

[52] Tramontano C., Miranda B., Chianese G., De Stefano L., Forestiere C., Pirozzi M., Rea I. (2021). Design of gelatin-capped plasmonic-diatomite nanoparticles with enhanced galunisertib loading capacity for drug delivery applications. Int. J. Mol. Sci..

[53] Thabet Y., Klingmann V., Breitkreutz J. (2018). Drug Formulations: Standards and Novel Strategies for Drug Administration in Pediatrics. J. Clin. Pharmacol..

[54] Glassman P.M., Muzykantov V.R. (2019). Pharmacokinetic and Pharmacodynamic Properties of Drug Delivery Systems. J. Pharmacol. Exp. Ther..

[55] Lombardo R., Musumeci T., Carbone C., Pignatello R. (2021). Nanotechnologies for Intranasal Drug Delivery: An Update of Literature. Pharm. Dev. Technol..

[56] Adeosun S.O., Ilomuanya M.O., Gbenebor O.P., Dada M.O., Odili C.C. (2020). Biomaterials for Drug Delivery: Sources, Classification, Synthesis, Processing, and Applications. Advanced Functional Materials.

[57] Hakim L.K., Yazdanian M., Alam M., Abbasi K., Tebyaniyan H., Tahmasebi E., Khayatan D., Seifalian A., Ranjbar R., Yazdanian A. (2021). Biocompatible and Biomaterials Application in Drug Delivery System in Oral Cavity. Evid. Based Complement. Altern. Med..

[58] Arif U., Haider S., Haider A., Khan N., Alghyamah A.A., Jamila N., Khan M.I., Almasry W.A., Kang I.-K. (2019). Biocompatible Polymers and Their Potential Biomedical Applications: A Review. Curr. Pharm. Des..

[59] Phogat S., Saxena A., Kapoor N., Aggarwal C., Tiwari A. (2021). Diatom Mediated Smart Drug Delivery System. J. Drug Deliv. Sci. Technol..

[60] Le T.D.H. (2021). Hydrophobic and hydrophilic drug loading capacity of micro diatom frustule from diatomite. J. Tech. Educ. Sci..

[61] Li M., Wu J., Lin D., Yang J., Jiao N., Wang Y., Liu L. (2022). A Diatom-Based Biohybrid Microrobot with a High Drug-Loading Capacity and PH-Sensitive Drug Release for Target Therapy. Acta Biomater..

[62] Delalat B., Sheppard V.C., Rasi Ghaemi S., Rao S., Prestidge C.A., McPhee G., Rogers M.L., Donoghue J.F., Pillay V., Johns T.G. (2015). Targeted Drug Delivery Using Genetically Engineered Diatom Biosilica. Nat. Commun..

[63] Delasoie J., Schiel P., Vojnovic S., Nikodinovic-Runic J., Zobi F. (2020). Photoactivatable Surface-Functionalized Diatom Microalgae for Colorectal Cancer Targeted Delivery and Enhanced Cytotoxicity of Anticancer Complexes. Pharmaceutics.

[64] Kumeria T., Bariana M., Altalhi T., Kurkuri M. (2013). Graphene Oxide Decorated Diatom Silica Particles as New Nano-Hybrids: Towards Smart Natural Drug Microcarriers. J. Mater. Chem. B..

[65] Bariana M., Aw M.S., Kurkuri M., Losic D. (2013). Tuning Drug Loading and Release Properties of Diatom Silica Microparticles by Surface Modifications. Int. J. Pharm..

[66] Vona D., Leone G., Ragni R., Palumbo F., Evidente A., Vurro M., Farinola G.M., Cicco S.R. (2016). Diatoms Biosilica as Efficient Drug-Delivery System. MRS Adv..

[67] Kabir A., Nazeer N., Bissessur R., Ahmed M. (2020). Diatoms Embedded, Self-Assembled Carriers for Dual Delivery of Chemotherapeutics in Cancer Cell Lines. Int. J. Pharm..

[68] Delasoie J., Rossier J., Haeni L., Rothen-Rutishauser B., Zobi F. (2018). Slow-Targeted Release of a Ruthenium Anticancer Agent from Vitamin B12 Functionalized Marine Diatom Microalgae. Dalton Trans..

[69] Aw M.S., Simovic S., Yu Y., Addai-Mensah J., Losic D. (2012). Porous Silica Microshells from Diatoms as Biocarrier for Drug Delivery Applications. Powder Technol..

[70] Saxena A., Dutta A., Kapoor N., Kumar A., Tiwari A. (2022). Envisaging Marine Diatom Thalassiosira Weissflogii as a SMART Drug Delivery System for Insoluble Drugs. J. Drug Deliv. Sci. Technol..

[71] Vona D., Flemma A., Piccapane F., Cotugno P., Cicco S.R., Armenise V., Vicente-Garcia C., Giangregorio M.M., Procino G., Ragni R. (2023). Drug Delivery through Epidermal Tissue Cells by Functionalized Biosilica from Diatom Microalgae. Mar. Drugs.

[72] Aw M.S., Simovic S., Addai-Mensah J., Losic D. (2011). Silica Microcapsules from Diatoms as New Carrier for Delivery of Therapeutics. Nanomedicine.

[73] Mancera-Andrade E.I., Parsaeimehr A., Ruiz-Ruiz F., Rorrer G.L., González-Valdez J., Iqbal H.M.N., Parra-Saldivar R. (2019). Isorhamnetin Encapsulation into Biogenic Silica from *Cyclotella* sp. Using a Microfluidic Device for Drug Delivery Applications. Biocatal. Agric. Biotechnol..

[74] Ibrahim S.M., Bin Jumah M.N., Othman S.I., Alruhaimi R.S., Al-Khalawi N., Salama Y.F., Allam A.A., Abukhadra M.R. (2021). Synthesis of Chitosan/Diatomite Composite as an Advanced Delivery System for Ibuprofen Drug; Equilibrium Studies and the Release Profile. Acs Omega.

[75] Le T.D.H., Bonani W., Speranza G., Sglavo V., Ceccato R., Maniglio D., Motta A., Migliaresi C. (2016). Processing and Characterization of Diatom Nanoparticles and Microparticles as Potential Source of Silicon for Bone Tissue Engineering. Mater. Sci. Eng. C.

[76] Luo Y., Li S., Shen K., Song Y., Zhang J., Su W., Yang X. (2021). Study on the Hemostasis Characteristics of Biomaterial Frustules Obtained from Diatom *Navicula australoshetlandica* sp.. Materials.

[77] Losic D., Yu Y., Aw M.S., Simovic S., Thierry B., Addai-Mensah J. (2010). Surface functionalisation of diatoms with dopamine modified iron-oxide nanoparticles: Toward magnetically guided drug microcarriers with biologically derived morphologies. Chem. Commun..

[78] Bariana M., Aw M.S., Losic D. (2013). Tailoring Morphological and Interfacial Properties of Diatom Silica Microparticles for Drug Delivery Applications. Adv. Powder Technol..

[79] Milović M., Simović S., Lošić D., Dashevskiy A., Ibrić S. (2014). Solid Self-Emulsifying Phospholipid Suspension (SSEPS) with Diatom as a Drug Carrier. Eur. J. Pharm. Sci..

[80] Uthappa U.T., Sriram G., Brahmkhatri V., Kigga M. (2018). Xerogel Modified Diatomaceous Earth Microparticles for Controlled Drug Release Studies. New J. Chem..

[81] Serhan M., Jackemeyer D., Long M., Sprowls M., Perez I.D., Maret W., Chen F., Tao N., Forzani E. (2020). Total iron measurement in human serum with a novel smartphone-based assay. J. Transl. Eng. Health Med..

[82] Javalkote V.S., Pandey A.P., Puranik P.R., Deshmukh P.K. (2015). Magnetically Responsive Siliceous Frustules for Efficient Chemotherapy. Mater. Sci. Eng. C.

[83] Cicco S.R., Vona D., Leone G., De Giglio E., Bonifacio M.A., Cometa S., Fiore S., Palumbo F., Ragni R., Farinola G.M. (2019). In Vivo Functionalization of Diatom Biosilica with Sodium Alendronate as Osteoactive Material. Mater. Sci. Eng. C.

[84] Cicco S.R., Vona D., Giglio E.D., Cometa S., Mattioli- M., Palumbo F., Ragni R., Farinola G.M. (2015). Chemically Modified Diatoms Biosilica for Bone Cell Growth with Combined Drug-Delivery and Antioxidant Properties. Chempluschem.

[85] Furman D., Campisi J., Verdin E., Carrera-Bastos P., Targ S., Franceschi C., Ferrucci L., Gilroy D.W., Fasano A., Miller G.W. (2019). Chronic Inflammation in the Etiology of Disease across the Life Span. Nat. Med..

[86] Delasoie J., Radakovic N., Pavic A., Zobi F. (2020). Neovascularization effects of carbon monoxide releasing drugs chemisorbed on coscinodiscus diatoms carriers characterized by spectromicroscopy imaging. Appl. Sci..

[87] Sasirekha R., Sheena T.S., Deepika M.S., Santhanam P., Townley H.E., Jeganathan K., Kumar S.D., Premkumar K. (2019). Surface Engineered Amphora Subtropica Frustules Using Chitosan as a Drug Delivery Platform for Anticancer Therapy. Mater. Sci. Eng. C.

[88] Wang L., Pan K., Li J., Li Y., Zhu B., Wang Y., Feng C., Han J. (2019). Influence of the Physicochemical Characteristics of Diatom Frustules on Hemorrhage Control. Biomater. Sci..

[89] Su C., Cao Z., Liu J., Sun X., Qiu K., Mu Y., Cong X., Wang X., Chen X., Jia N. (2023). The Hierarchical Porous Structures of Diatom Biosilica-Based Hemostat: From Selective Adsorption to Rapid Hemostasis. J. Colloid Interface Sci..

[90] van den Boogaard W.M.C., Komninos D.S.J., Vermeij W.P. (2022). Chemotherapy Side-Effects: Not All DNA Damage Is Equal. Cancers.

[91] Anand U., Dey A., Chandel A.K.S., Sanyal R., Mishra A., Pandey D.K., de la Lastra J.M.P. (2023). Cancer chemotherapy and beyond: Current status, drug candidates, associated risks and progress in targeted therapeutics. Genes Dis..

[92] Pandya P., Giram P., Bhole R.P., Chang H.I., Raut S.Y. (2021). Nanocarriers based oral lymphatic drug targeting: Strategic bioavailability enhancement approaches. J. Drug Deliv. Sci. Technol..

[93] Johnson L.T., Zhang D., Zhou K., Lee S.M., Liu S., Dilliard S.A., Farbiak L., Chatterjee S., Lin Y.H., Siegwart D.J. (2022). Lipid Nanoparticle (LNP) Chemistry Can Endow Unique in Vivo RNA Delivery Fates within the Liver That Alter Therapeutic Outcomes in a Cancer Model. Mol. Pharm..

[94] Duan H., Liu Y., Gao Z., Huang W. (2021). Recent Advances in Drug Delivery Systems for Targeting Cancer Stem Cells. Acta Pharm. Sin. B.

[95] Li Y., Lv W., Wang L., Zhang Y., Yang L., Wang T., Zhu L., Wang Y., Wang W. (2021). Photo-Triggered Nucleus Targeting for Cancer Drug Delivery. Nano Res..

[96] Uthappa U.T., Kigga M., Sriram G., Ajeya K.V., Jung H.-Y., Neelgund G.M., Kurkuri M.D. (2019). Facile Green Synthetic Approach of Bio Inspired Polydopamine Coated Diatoms as a Drug Vehicle for Controlled Drug Release and Active Catalyst for Dye Degradation. Microporous Mesoporous Mater..

[97] Senapati S., Shukla R., Tripathi Y.B., Mahanta A.K., Rana D., Maiti P. (2018). Engineered Cellular Uptake and Controlled Drug Delivery Using Two Dimensional Nanoparticle and Polymer for Cancer Treatment. Mol. Pharm..

[98] Maleki A., Kettiger H., Schoubben A., Rosenholm J.M., Ambrogi V., Hamidi M. (2017). Mesoporous Silica Materials: From Physico-Chemical Properties to Enhanced Dissolution of Poorly Water-Soluble Drugs. J. Control. Release.

[99] Mehmood Y., Khan I.U., Shahzad Y., Khan R.U., Iqbal M.S., Khan H.A., Khalid I., Yousaf A.M., Khalid S.H., Asghar S. (2020). In Vitro and in Vivo Evaluation of Velpatasvir-Loaded Mesoporous Silica Scaffolds. A Prospective Carrier for Drug Bioavailability Enhancement. Pharmaceutics.

[100] Tesson B., Genet M.J., Fernandez V., Degand S., Rouxhet P.G., Martin-Jézéquel V. (2009). Surface Chemical Composition of Diatoms. ChemBioChem.

[101] Rogato A., De Tommasi E. (2020). Physical, chemical, and genetic techniques for diatom frustule modification: Applications in nanotechnology. Appl. Sci..

[102] Placha D., Jampilek J. (2021). Chronic inflammatory diseases, anti-inflammatory agents and their delivery nanosystems. Pharmaceutics.

[103] Newton K., Dixit V.M., Kayagaki N. (2021). Dying Cells Fan the Flames of Inflammation. Science.

[104] Dinarello C.A. (2010). Review Anti-Inflammatory Agents Present and Future. Cell.

[105] Luo X., Matranga C., Tan S., Alba N., Cui X.T. (2011). Carbon Nanotube Nanoreservior for Controlled Release of Anti-Inflammatory Dexamethasone. Biomaterials.

[106] Steinberg D., Friedman M. (2017). Sustained-Release Drug Delivery of Antimicrobials in Controlling of Supragingival Oral Biofilms. Expert Opin. Drug Deliv..

[107] Yang S., Han X., Yang Y., Qiao H., Yu Z., Liu Y., Wang J., Tang T. (2018). Bacteria-targeting nanoparticles with microenvironment-responsive antibiotic release to eliminate intracellular Staphylococcus aureus and associated infection. ACS Appl. Mater. Interfaces.

[108] Peng G., Cai J., Wang Z., Zhang W., Xu J., Zhang D., Gong D. (2024). Facile Fabrication of Diatomite Biosilica-Based Nasal Drug Delivery Vehicle for Enhanced Treatment of Allergic Rhinitis. Colloids Surf. B Biointerfaces.

[109] Kushioka J., Kwoon S., Chow H., Toya M., Tsubosaka M., Shen H. (2023). Bone Regeneration in Inflammation with Aging and Cell—Based Immunomodulatory Therapy. Inflamm. Regen..

[110] Jang J.-W., Min K.-E., Kim C., Shin J., Lee J., Yi S. (2023). Scaffold Characteristics, Fabrication Methods, and Biomaterials for the Bone Tissue Engineering. Int. J. Precis. Eng. Manuf..

[111] Abbas M., Alqahtani M.S., Alhifzi R. (2023). Recent developments in polymer nanocomposites for bone regeneration. Int. J. Mol. Sci..

[112] Gu W., Wu C., Chen J., Xiao Y. (2013). Bone Diseases and Bone Regeneration Nanotechnology in the Targeted Drug Delivery for Bone Diseases and Bone Regeneration. Int. J. Nanomed..

[113] Newman M.R., Benoit D.S.W. (2016). ScienceDirect Local and Targeted Drug Delivery for Bone Regeneration. Curr. Opin. Biotechnol..

[114] El-Husseiny H.M., Mady E.A., El-Dakroury W.A., Zewail M.B., Noshy M., Abdelfatah A.M., Doghish A.S. (2022). Smart/Stimuli-Responsive Hydrogels: State-of-the-Art Platforms for Bone Tissue Engineering. Appl. Mater. Today.

[115] Municoy S., Alvare Echazu M.I., Antezana P.E., Galdoporpora J.M., Olivetti C., Mebert A.M., Foglia M.L., Tuttolomondo M.V., Alvarez G.S., Hardy J.G. (2020). Stimuli-responsive materials for tissue engineering and drug delivery. Int. J. Mol. Sci..

[116] Kwon S., Singh R.K., Perez R.A., Abou Neel E.A., Kim H.W., Chrzanowski W. (2013). Silica-based mesoporous nanoparticles for controlled drug delivery. J. Tissue Eng..

[117] Li X., Sun Q., Li Q., Kawazoe N., Chen G. (2018). Functional Hydrogels With Tunable Structures and Properties for Tissue Engineering Applications. Front. Chem.

[118] Dalgic A.D., Atila D., Tezcaner A., Gürses S., Keskin D. (2023). Diatom Silica Frustules-Doped Fibers for Controlled Release of Melatonin for Bone Regeneration. Eur. Polym. J..

[119] Mao L., Xia L., Chang J., Liu J., Jiang L., Wu C., Fang B. (2017). The Synergistic Effects of Sr and Si Bioactive Ions on Osteogenesis, Osteoclastogenesis and Angiogenesis for Osteoporotic Bone Regeneration. Acta Biomater..

[120] Lu X., Yu S., Chen G., Zheng W., Peng J., Huang X., Chen L. (2021). Insight into the Roles of Melatonin in Bone Tissue and Bone-Related Diseases. Int. J. Mol. Med..

[121] Xiao L., Lin J., Chen R., Huang Y., Liu Y., Bai J., Ge G., Shi X., Chen Y., Shi J. (2020). Sustained Release of Melatonin from GelMA Liposomes Reduced Osteoblast Apoptosis and Improved Implant Osseointegration in Osteoporosis. Oxidative Med. Cell. Longev..

[122] Mohammadi M., Abbaszadeh S., Nosrati-siahmazgi V. (2024). Heliyon Diatom-Guided Bone Healing via a Hybrid Natural Scaffold. Heliyon.

[123] Eming S.A., Krieg T., Davidson J.M., Hall R.P. (2007). Inflammation in Wound Repair: Molecular and Cellular Mechanisms. J. Investig. Dermatol..

[124] Leaper D.J., Schultz G., Carville K., Fletcher J., Swanson T., Drake R. (2012). Extending the TIME concept: What have we learned in the past 10 years?. Int. Wound J..

[125] Shaw T.J., Martin P. (2009). Wound Repair at a Glance. J. Cell Sci..

[126] Flynn K., Mahnoud N.N., Sharifi S., Gould L.J., Mahmoudi M. (2023). Chronic wound healing models. ACS Pharmacol. Transl. Sci..

[127] Qiu W., Han H., Li M., Li N., Wang Q., Qin X., Wang X., Yu J., Li Y., Li F. (2021). Nanofibers Reinforced Injectable Hydrogel with Self-Healing, Antibacterial, and Hemostatic Properties for Chronic Wound Healing. J. Colloid Interface Sci..

[128] Wang Y., Wu Y., Long L., Yang L., Fu D., Hu C., Wang Y., Kong Q. (2021). Inflammation-responsive drug-loaded hydrogels with sequential hemostasis, antibacterial, and anti-inflammatory behavior for chronically infected diabetic wound treatment. ACS Appl. Mater. Interfaces.

[129] Liu L., Hu E., Yu K., Xie R., Lu F., Lu B., Bao R., Li Q., Dai F., Lan G. (2021). Recent Advances in Materials for Hemostatic Management. Biomater. Sci..

[130] Wang C., Niu H., Ma X., Hong H., Yuan Y., Liu C. (2019). Bioinspired, injectable, quaternized hydroxyethyl cellulose composite hydrogel coordinated by mesocellular silica foam for rapid, noncompressible hemostasis and wound healing. ACS Appl. Mater. Interfaces.

[131] Sun X., Li N., Su C., Mu Y., Cong X., Cao Z., Wang H., Yang X., Chen X., Feng C. (2023). Diatom-inspired bionic hydrophilic polysaccharide adhesive for rapid sealing hemostasis. ACS Nano..

[132] Khraisheh M.A.M., Al-Ghouti M.A., Allen S.J., Ahmad M.N. (2005). Effect of OH and silanol groups in the removal of dyes from aqueous solution using diatomite. Water Res..

[133] Townley H.E., Parker A.R., White-cooper H. (2008). Exploitation of diatom frustules for nanotechnology: Tethering active biomolecules. Adv. Funct. Mater..

[134] Kobayashi M., Juillerat F., Galletto P., Bowen P., Borkovec M. (2005). Aggregation and charging of colloidal silica particles: Effect of particle size. Langmuir.

[135] Hassanali A.A., Zhang H., Knight C., Shin Y.K., Singer S.J. (2010). The dissociated amorphous silica surface: Model development and evaluation. J. Chem. Theory Comput..

[136] Aw M.S., Bariana M., Yu Y., Addai-Mensah J., Losic D. (2013). Surface-functionalized diatom microcapsules for drug delivery of water-insoluble drugs. J. Biomater. Appl..

[137] Esfandyari J., Shojaedin-givi B., Hashemzadeh H., Mozafari-nia M. (2020). Photodiagnosis and Photodynamic Therapy Capture and Detection of Rare Cancer Cells in Blood by Intrinsic Fl Uorescence of a Novel Functionalized Diatom. Photodiagnosis Photodyn. Ther..

[138] Rozan H.E., Wu G., Zhou Z., Li Q., Sharaf M., Chen X. (2022). Colloids and Surfaces B Biointerfaces The Complex Hydrogel Based on Diatom Biosilica and Hydroxybutyl Chitosan for Wound Healing. Colloids Surf. B Biointerfaces.

[139] Cao Z., Wang X., Jiang C., Wang H., Mu Y., Sun X., Chen X., Feng C. (2024). Thermo-Sensitive Hydroxybutyl Chitosan/Diatom Biosilica Hydrogel with Immune Microenvironment Regulatory for Chronic Wound Healing. Int. J. Biol. Macromol..

[140] Lee J., Park E., Fujisawa A., Lee H. (2021). Diatom silica/polysaccharide elastomeric hydrogels: Adhesion and interlocking synergy. ACS Appl. Mater. Interfaces.

[141] Cao Z., Su C., Sun X., Shao K., Wang X., Mu Y., Chen X., Feng C. (2022). Enhanced Mechanical Properties of Hydroxybutyl Chitosan Hydrogel through Anchoring Interface Effects of Diatom Biosilica. Carbohydr. Polym..

[142] Wang Y., Fu Y., Li J., Mu Y., Zhang X., Zhang K., Liang M., Feng C., Chen X. (2018). Multifunctional Chitosan/Dopamine/Diatom-Biosilica Composite Beads for Rapid Blood Coagulation. Carbohydr. Polym..

[143] Hate S.S., Reutzel-Edens S.M., Taylor L.S. (2020). Influence of drug–silica electrostatic interactions on drug release from mesoporous silica-based oral delivery systems. Mol. Pharm..

[144] Todd T., Zhen Z., Tang W., Chen H., Wang G., Chuang Y.J., Xie J. (2014). Iron oxide nanoparticle encapsulated diatoms for magnetic delivery of small molecules to tumors. Nanoscale.

[145] Ooi Y.J., Wen Y., Zhu J., Song X., Li J. (2020). Surface charge switchable polymer/DNA nanoparticles responsive to tumor extracellular ph for tumor-triggered enhanced gene delivery. Biomacromolecules.

[146] Wu W., Wang J., Lin Z., Li X., Li J. (2014). Tumor-Acidity Activated Surface Charge-Conversion of Polymeric Nanocarriers for Enhanced Cell Adhesion and Targeted Drug Release. Macromol. Rapid Commun..

[147] Xu L., Wang X., Liu Y., Wang G., Falconer R.G., Zhao C.X. (2022). Lipid nanoparticles for drug delivery. Adv. NanoBiomed Res..

[148] Seo Y., Lim H., Park H., Yu J., An J., Yoo H.Y., Lee T. (2023). Recent Progress of Lipid Nanoparticles-Based Lipophilic Drug Delivery: Focus on Surface Modifications. Pharmaceutics.

[149] Yue Z.G., Wei W., Lv P.P., Yue H., Wang L.Y., Su Z.G., Ma G.H. (2011). Surface charge affects cellular uptake and intracellular trafficking of chitosan-based nanoparticles. Biomacromolecules.

[150] Sharma V., Anandhakumar S., Sasidharan M. (2015). Self-Degrading Niosomes for Encapsulation of Hydrophilic and Hydrophobic Drugs: An Efficient Carrier for Cancer Multi-Drug Delivery. Mater. Sci. Eng. C.

[151] Mirchandani Y., Patravale V.B., Brijesh S. (2021). Solid Lipid Nanoparticles for Hydrophilic Drugs. J. Control. Release.

[152] Herdiana Y., Wathoni N., Shamsuddin S., Joni I.M., Muchtaridi M. (2021). Chitosan-based nanoparticles of targeted drug delivery system in breast cancer treatment. Polymers.

[153] Tezgel Ö., Szarpark-Jankowska A., Arnould A., Auzely-Velty R., Texier I. (2018). Chitosan-lipid nanoparticles (CS-LNPs): Application to siRNA delivery. J. Colloid Interface Sci..

[154] Lv J., Sun B., Jin J., Jiang W. (2019). Mechanical and Slow-Released Property of Poly (Acrylamide) Hydrogel Reinforced by Diatomite. Mater. Sci. Eng. C.

[155] Nie J., Pei B., Wang Z., Hu Q. (2019). Construction of Ordered Structure in Polysaccharide Hydrogel A Review. Carbohydr. Polym..

[156] Jonker A.M., Löwik D.W., Van Hest C.M.J. (2012). Peptide-and protein-based hydrogels. Chem. Mater..

[157] Ghasemiyeh P., Mohammadi-Samani S. (2019). Hydrogels as Drug Delivery Systems; Pros and Cons. Trends Pharm. Sci..

[158] Zhang Y.S., Khademhosseini A. (2017). Advances in Engineering Hydrogels. Science.

[159] Larrañeta E., Henry M., Irwin N.J., Trotter J., Perminova A.A., Donnelly R.F. (2018). Synthesis and Characterization of Hyaluronic Acid Hydrogels Crosslinked Using a Solvent-Free Process for Potential Biomedical Applications. Carbohydr. Polym..

[160] Narayanaswamy R., Torchilin V.P. (2020). Hydrogels and their applications in targeted drug delivery. The Road from Nanomedicine to Precision Medicine.

[161] Qin P., Tang J., Sun D., Yang Y., Liu N., Li Y., Fu Z., Wang Y., Li C., Li X. (2022). Zn2+ cross-linked alginate carrying hollow silica nanoparticles loaded with RL-QN15 peptides provides promising treatment for chronic skin wounds. ACS Appl. Mater. Interfaces.

